# Mitochondrial Genome Analysis of *Babesia ovis* (Apicomplexa: Babesiidae) Endemic in Sheep in Türkiye

**DOI:** 10.3390/vetsci11110554

**Published:** 2024-11-10

**Authors:** Mehmet Can Ulucesme, Munir Aktas, Sezayi Ozubek

**Affiliations:** Department of Parasitology, Faculty of Veterinary Medicine, University of Fırat, Elazig 23200, Türkiye; mculucesme@firat.edu.tr (M.C.U.); maktas@firat.edu.tr (M.A.)

**Keywords:** *Babesia ovis*, mitochondrial genome, phylogenetic, evolutionary biology, ovine babesiosis

## Abstract

This study presents the first comprehensive analysis of the mitochondrial genome of *Babesia ovis*, a significant pathogen in ovine babesiosis in Türkiye. The *B. ovis* mitochondrial genome is a linear monomeric molecule of 6015 bp with an A + T content of 70.5%, featuring terminal inverted repeats (TIRs) at both ends. It encodes three essential proteins (Cox1, Cox3, and Cob) and six fragments of *large subunit rRNA* genes. Comparative analysis revealed high sequence identity with *Babesia* sp. Xinjiang (87.5%) and *Babesia* sp. Dunhuang (87.5%), suggesting a close evolutionary relationship. The study highlights the conservation of mitochondrial gene content across *Babesia* and *Theileria* species, emphasizing their utility in phylogenetic studies. The findings also suggest that TIR variability plays a role in mitochondrial genome size differences, influencing species-specific adaptations. This research provides valuable insights into the evolution and functional adaptations of *B. ovis* and underscores the potential of mitochondrial genomic data in enhancing diagnostic and therapeutic strategies for babesiosis. Further exploration of mitochondrial genomes in *Babesia* species is essential for understanding their biology and developing effective control measures.

## Simple Summary

This study analyzes the mitochondrial genome of *Babesia ovis*, a key pathogen in sheep babesiosis in Türkiye. The genome is a linear molecule of 6015 base pairs, rich in A + T content (70.5%), and contains terminal inverted repeats (TIRs). It encodes three proteins (Cox1, Cox3, and Cob) and six *large subunit rRNA* gene fragments. The genome shows high similarity (87.5%) to *Babesia* species from Xinjiang and Dunhuang, indicating a close evolutionary link. The research highlights the conserved mitochondrial genes across *Babesia* and *Theileria* species and suggests that TIR variability influences genome size and species adaptations. These findings could improve diagnostic and treatment methods for babesiosis, stressing the need for further research on *Babesia* mitochondrial genomes.

## 1. Introduction

Mitochondria are vital organelles within cells, playing key roles in energy production, metabolic processes, calcium regulation, and cellular signaling. Beyond their fundamental biological functions, mitochondrial genomes provide significant insights into the genetic evolution, classification, and biological characteristics of protozoans. Research across various phylogenetic groups has uncovered considerable diversity in the size, structure, and organization of mitochondrial genomes [1,2,3,4,5].

The mitochondrial genome, found in nearly all eukaryotic cells, displays a remarkable range of variability. Typically, animal mitochondrial genomes are circular, spanning 15 kb to 20 kb, and encompass *12–13 protein-coding* genes, *22 transfer RNA (tRNA)* genes, and *two ribosomal RNA (rRNA)* genes, with relatively conserved gene arrangements [6]. However, linear forms of mitochondrial genomes have been identified in many apicomplexan parasites, such as *Plasmodium*, *Babesia*, *Theileria*, and *Eimeria*, generally measuring around 6 kb [5,7,8]. These apicomplexan mitochondrial genomes typically encode only three protein-coding genes—*cytochrome c oxidase subunits I* (*cox1*) and *III* (*cox3*) and *cytochrome b* (*cob*)—alongside six fragmented *large subunit rRNA* genes [7,9]. In contrast to animal mitochondrial genomes, apicomplexan mitochondrial genomes do not include tRNAs or other essential translation components, which are encoded by the nuclear genome and imported into mitochondria from the cytosol [10,11]. This distinctive genomic organization makes mitochondrial protein-coding genes in apicomplexan parasites ideal for studies on phylogenetics, gene rearrangement, and evolutionary biology [2,7].

Investigating mitochondrial genomes can yield new perspectives on the biological traits, genetic evolution, and classification of apicomplexan parasites, as well as contribute valuable data for developing anti-*Babesia* treatment. While the mitochondrial genomes of several *Babesia* species, such as *Babesia bigemina*, *B. bovis*, *B. caballi*, *B. canis*, *B. conradae*, *B. duncani*, *B. gibsoni*, *B. microti*, *B. orientalis*, *B. rodhaini*, *B. rossi*, *B. vogeli*, and various genotypes of *B. motasi*—along with those of *Theileria equi*, *T. orientalis*, and *T. luwenshuni*—have been sequenced; limited information is available on the mitochondrial genomes of *B. ovis*.

Globally, more than 100 *Babesia* species have been identified that infect humans, as well as domestic and wild mammals and birds. This number is expected to rise as further research explores additional vertebrate hosts [12,13]. These parasites cause a disease characterized by symptoms such as high fever, anemia, jaundice, and hemoglobinuria, and in severe cases, can lead to death, particularly in domestic ruminants [14]. Ovine babesiosis is a significant concern for small ruminant health, with *Babesia ovis*, *B. motasi*, and *B. crassa* being the primary culprits of babesiosis in these animals [12]. Among them, *B. ovis* is the predominant pathogen responsible for clinical disease in sheep [15].

As *B. ovis* is a significant pathogen affecting sheep, particularly in Türkiye and neighboring regions [16,17], studying its mitochondrial genome is crucial for understanding its unique biological and evolutionary characteristics. This species may harbor distinct mitochondrial features that can provide new insights into the evolution of mitochondrial genomes among apicomplexan parasites. Furthermore, characterizing the mitochondrial genome of *B. ovis* can aid in identifying species-specific molecular markers, enhancing diagnostic accuracy and epidemiological tracking. These insights are essential for developing targeted treatments and control strategies against ovine babesiosis, a disease with a substantial economic impact on the livestock industry [5,7,18].

Moreover, the identification of new molecular markers derived from mitochondrial genomes can significantly enhance the diagnosis and classification of *Babesia* species. Mitochondrial genes such as Cox1 and Cob are highly conserved yet contain enough genetic variation to differentiate between species, making them excellent targets for molecular diagnostics [7]. These markers improve the sensitivity and specificity of PCR-based diagnostic assays, facilitating the detection and differentiation of *Babesia* infections in clinical samples [5,19]. Additionally, mitochondrial genome data provide valuable phylogenetic signals that can resolve taxonomic ambiguities among closely related *Babesia* species [4]. By expanding the repertoire of molecular markers, our study contributes to more accurate epidemiological surveillance and control of babesiosis [5,19]. Despite the use of the *18S rRNA* gene in diagnosing *Babesia* infections, accurately classifying species within this genus remains challenging due to high sequence similarity among closely related species. For example, *B. divergens* and *B. capreoli*, which infect different hosts, have nearly identical *18S rRNA* sequences, differing by only a few nucleotides [20,21]. This high similarity can lead to misidentification or synonymization of species. Mitochondrial genes like cox1 offer greater genetic diversity and specificity, making them more effective for distinguishing *Babesia* species and clarifying phylogenetic relationships [4,7,22]. Sequencing mitochondrial genomes provides valuable genetic data that can resolve taxonomic ambiguities, reveal cryptic diversity, and aid in the identification of new species [23,24,25,26]. These factors highlight the importance of mitochondrial genome sequencing in enhancing species classification and deepening our understanding of the evolutionary history of *Babesia* species [4].

This study aims to address this gap by focusing on the mitochondrial genome of *B. ovis*, an apicomplexan parasite prevalent in sheep in Türkiye [27,28,29,30]. Through sequencing and analyzing the *B. ovis*-Alacakaya strain, we seek to clarify its phylogenetic relationships and classification within the *Babesia* genus. The sequences obtained were assembled, annotated, compared with those of other piroplasms, and submitted to GenBank. By providing the first comprehensive analysis of the *B. ovis* mitochondrial genome, we offer valuable insights into its unique genetic makeup and evolutionary position. This research offers valuable insights into the evolution of mitochondrial genomes among apicomplexan parasites and identifies new molecular markers that could enhance the diagnosis and classification of *Babesia* species. Furthermore, these findings have the potential to inform drug target screening and the development of effective treatments for babesiosis in livestock.

## 2. Materials and Methods

### 2.1. Parasites and Isolation of Genomic DNA

Genomic DNA was extracted from *B. ovis*-Alacakaya stabilate obtained from a splenectomized sheep that was experimentally infected [15]. The extraction process utilized 200 µL of EDTA-anticoagulated blood from the sheep, employing the PureLink™ Genomic DNA Mini Kit (Invitrogen Corporation, Carlsbad, CA, USA), following the manufacturer’s protocol. Before sequencing, the quality and quantity of extracted DNA were assessed using a NanoDrop spectrophotometer (Thermo Fisher Scientific, Waltham, MA, USA) to measure the A260/A280 ratio, ensuring values between 1.8 and 2.0, which indicates high purity. Additionally, DNA integrity was checked by running samples on a 1% agarose gel, where the presence of a distinct high-molecular-weight band confirmed that the DNA was intact and suitable for sequencing.

### 2.2. Amplification of B. ovis Mitochondrial Genome and Sequence Analyzing

PCR primers were designed based on the genomic sequences available for *B. bovis* (AB499088.1), *B. bigemina* (AB499085.1), *B. orientalis* (KF218819.1), *B. caballi* (AB499086.1), and *B. gibsoni* (AB499087.1). The primers were generated using the Primer Quest™ Tool “https://www.idtdna.com/pages/tools/primerquest (accessed on: 1 June 2024)” (Supplementary Figure S1). PCR amplification was carried out using the Phusion^®^ High-Fidelity PCR Master Mix with GC Buffer (#M0532S; NEB, Ipswich, MA, USA) in a reaction volume of 20 µL, which included 10 µL of 2× Phusion Master Mix, 1 µL each of forward and reverse primers (10 pmol/μL), 1 µL of template DNA (approximately 20 ng/µL), and 7 µL of nuclease-free water. The complete mitochondrial genome was amplified using primer combinations (Table 1). The PCR was performed with the following cycling conditions: an initial denaturation step at 98 °C for 30 s, followed by 35 cycles of denaturation at 98 °C for 30 s, annealing at 55 °C for 30 s, and extension at 72 °C for 1.5 min. A final extension step was carried out at 72 °C for 5 min. The PCR products were then subjected to Sanger sequencing for mitochondrial genome determination. The sequencing library was prepared using Nextera XT DNA Library Preparation Kit, and sequencing was performed by Illumina Miseq platform as paired-end (PE) 2 × 150 bases reads. Raw NGS reads (FASTQ) were quality-checked by FASTQC [31] and trimmed by Trimmomatic v0.32 [32]. Demultiplexing and low-quality read filtering were performed via CLC Genomics Workbench (Qiagen, Hilden, Germany). The de novo assembly constructed by CLC Genomic’s de novo assembly module with the parameters of minimum contig size as 200 bp, mismatch cost is 2, insert cost is 3, deletion cost is 3, length fraction is 0.5, similarity fraction is 0.8, and paired read input as minimum distance of 180 (FicusBio, Ankara, Türkiye).

The nucleotide sequences of the *B. ovis* mitochondrial genome (GenBank accession no. PP973837) were aligned with published mitochondrial genome sequences from *B. bovis* (AB499088.1), *B. bigemina* (AB499085.1), *B. orientalis* (KF218819.1), *B. caballi* (AB499086.1), and *B. gibsoni* (AB499087.1) using the Clustal Omega Multiple Alignment tool “http://www.ebi.ac.uk/Tools/msa/clustalo (1 March 2024)”. Protein-coding genes were annotated by comparison with previously annotated sequences from these five *Babesia* species. Putative rRNA genes were identified by comparing mitochondrial DNA sequences or annotated rRNA gene fragments from these species using pairwise Blast comparisons on the NCBI platform. Phylogenetic tree construction was performed using the MEGA11 software version 11.0.13 [33].

## 3. Results

The mitochondrial genome of *B. ovis* is a linear, monomeric molecule with a total length of 6015 bp and an A + T content of 70.5%, featuring terminal inverted repeats (TIRs) at both ends. This genome encodes three essential genes: *cox1* (1434 bp), *cob* (1092 bp), and *cox3* (639 bp), along with six fragments of the *large subunit* (*LSU*) *rRNA* genes. The lengths of these rRNA fragments are 303 bp (*LSU1*), 111 bp (*LSU3*), 38 bp (*LSU6*), 36 bp (*LSU2*), 68 bp (*LSU5*), and 82 bp (*LSU4*).

In the mitochondrial genome of *B. ovis*, 117 bp TIRs were identified at both the 5′ and 3′ ends of the project numbered 222O123. BLAST analysis of the complete mitochondrial genome sequence of *B. ovis* against the NCBI database revealed a high degree of sequence similarity with other *Babesia* species. The *B. ovis* mitochondrial genome shares 87.5% identity with *Babesia* sp. Xinjiang (MK962313.1) and *Babesia* sp. Dunhuang (MK962314.1), 86.6% identity with *B. caballi* (AB499086.1), 86.2% with *B. bovis* (NC_009902.1), and 85.3% with multiple genotypes of *B. motasi* (Hebei, MN605892.1; Ningxian, MN605891.1; Tianzhu, MN605890.1; Lintan, MN605889.1). Further comparisons show 84.8% identity with *B. bigemina* (AB499085.1), 83.6% with *B. gibsoni* (AB499087.1), 83.8% with *T. uilenbergi* (MZ231018.1), and 79.7% with *T. lestoquardi* (NC_053925.1).

**Figure 1:**
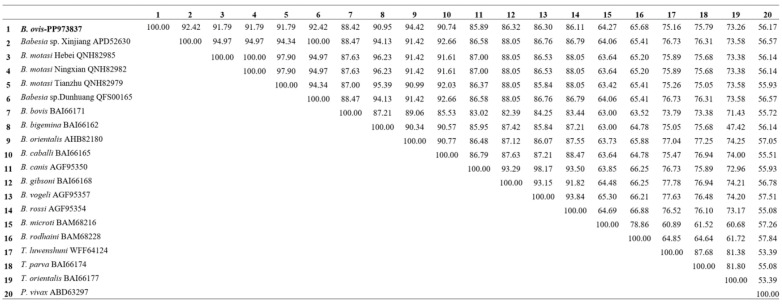
Comparative sequence identity matrix of the Cox1 protein across *B. ovis* (bold) and related apicomplexan species.

**Figure 2:**
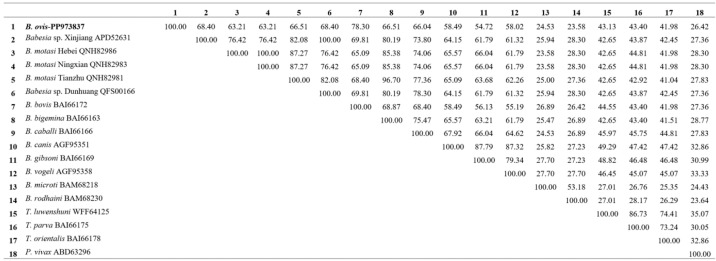
Comparative sequence identity matrix of the Cox3 protein across *B. ovis* (bold) and related apicomplexan species.

**Figure 3:**
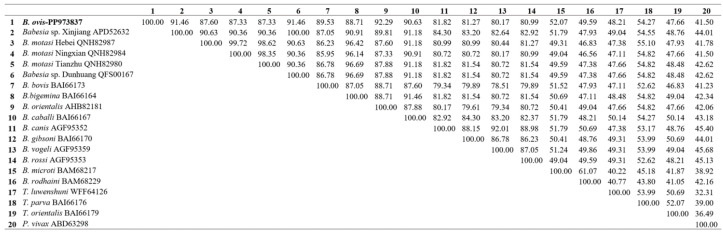
Comparative sequence identity matrix of the Cob protein across *B. ovis* (bold) and related apicomplexan species.

The cox1 protein in *B. ovis* showed high sequence identity with *Babesia* sp. Xinjiang (92.42%) and *B. motasi* Hebei (91.79%), indicating a close evolutionary relationship. The sequence identity with *B. bovis* and *B. caballi* was slightly lower, at 88.42% and 90.74%, respectively. The lowest identity was observed with *P. vivax* at 56.17%, reflecting the more distant relationship between these species (Figure 1). For the cox3 protein, *B. ovis* showed high sequence identity with *B. bovis* (78.30%), *Babesia* sp. Xinjiang (68.40%) and *Babesia* sp. Dunhuang (68.40%), indicating a close evolutionary relationship among these species. The sequence identity with *B. vogeli* and *B. gibsoni* was slightly lower, at 58.02% and 54.72%, respectively. The lowest identity was observed with *B. rodhaini* (23.58%) and *B. microti* (24.53%), reflecting the more distant evolutionary relationship between these species (Figure 2). For the cob protein, *B. ovis* showed high sequence identity with *Babesia orientalis* (92.29%), *Babesia* sp. Xinjiang (91.46%), and *Babesia* sp. Dunhuang (91.46%), indicating a close evolutionary relationship among these species. The sequence identity with *B. vogeli* and *B. rossi* was slightly lower, at 80.17% and 80.99%, respectively. The lowest identity was observed with *P. vivax* (41.50%) and *Theileria orientalis* (47.66%), reflecting the more distant evolutionary relationship between these species (Figure 3).

**Figure 4:**
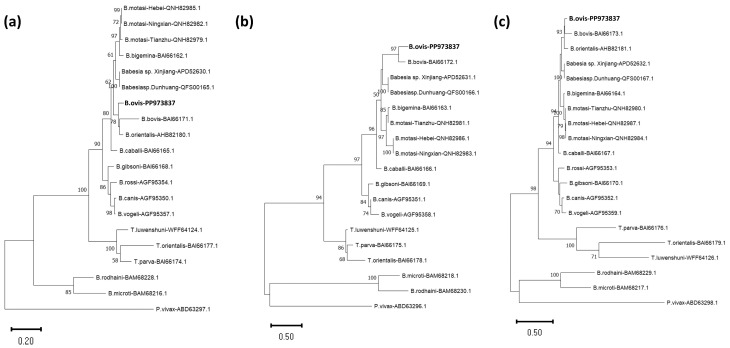
Phylogenetic trees of *B. ovis* (in bold) and related apicomplexan species based on amino acid sequences of (**a**) Cox1, (**b**) Cox3, and (**c**) Cob proteins. The trees were constructed using the Maximum Likelihood method in MEGA11, illustrating the evolutionary relationships among species within the Piroplasmida order. Evolutionary analyses were performed using the Le_Gascuel_2008 model [34] for cox1 and cob and the Jones et al. w/freq. model [35] for cox3. Bootstrap values (from 100 replicates) are indicated at branch nodes, with values above 50% displayed. GenBank accession numbers are provided next to species names. The scale bar represents nucleotide substitutions per site, indicating evolutionary distances.

The phylogenetic analysis of *B. ovis* based on the cox1, cox3, and cob amino acid sequences reveals its close evolutionary relationship with *B. bovis*, *B. orientalis*, *Babesia* sp. Xinjiang and *Babesia* sp. Dunhuang (Figure 4). In all three phylogenetic trees, *B. ovis* forms a well-supported clade distinctly separated from other *Babesia* species, such as *B. bigemina* and *B. motasi*. This analysis highlights the genetic divergence of *B. ovis* within the *Babesia* genus while confirming its close ties with certain related species. The findings provide important insights into the evolutionary positioning of *B. ovis* among apicomplexan parasites.

In terms of gene content, the *cox1* gene in *B. ovis* is 1434 bp long, identical to that in *B. bovis*, *B. bigemina*, and other related species. The *cox3* gene of *B. ovis* is 639 bp, consistent with the sizes found in other *Babesia* species. The *large subunit* (*LSU*) *rRNA* gene fragments in *B. ovis* vary in length, with *LSU1* being 303 bp, *LSU3* at 111 bp, and *LSU6* at 38 bp, aligning closely with the corresponding genes in other *Babesia* species. The *cob* gene (cytochrome b) in *B. ovis* is 1092 bp, identical across the compared *Babesia* species (Table 2).

## 4. Discussion

This study represents the first comprehensive analysis of the mitochondrial genome of *B. ovis*, a pathogen of significant concern in ovine babesiosis, particularly in Türkiye. The findings provide critical insights into the genetic makeup, structure, and potential evolutionary adaptations of *B. ovis* within the broader context of *Babesia* and *Theileria* species.

Terminal inverted repeats (TIRs) are an important structural element found across various *Babesia* species, and they play a critical role in the replication and stabilization of linear mitochondrial genomes [22]. The variability in TIR length between species, such as the shorter TIRs observed in *B. caballi* compared to the more complex TIR systems in *B. microti* and *B. rodhaini*, may reflect evolutionary adaptations to different host environments or life cycle requirements [5,7,8]. For instance, species with longer and more complex TIR structures, like *B. microti*, might require additional genomic stability mechanisms due to their more diverse or complex host interactions and environmental challenges. The presence of shorter TIRs in *B. ovis* and related species may indicate that they have evolved streamlined mechanisms for genome replication and stability, which could be linked to their specific ovine host and its ecological niche. The variability in TIR length across different species is likely a key factor contributing to differences in mitochondrial genome sizes among apicomplexans. For example, *B. microti* and *B. rodhaini* possess a dual flip-flop inversion system [8], which adds to their mitochondrial genome length, whereas other species like *B. caballi* and various genotypes of *B. motasi* exhibit shorter and simpler TIR structures [5].

The high degree of sequence identity (87.5%) between the *B. ovis* mitochondrial genome and that of *Babesia* sp. Xinjiang and *Babesia* sp. Dunhuang suggests a close evolutionary relationship. This close similarity raises intriguing questions about the evolutionary pathways and geographical distribution of these *Babesia* species. It also points to the possibility of shared or similar host species, ecological niches, or transmission vectors in these regions. The slightly lower sequence identity observed with *B. bovis* (86.2%) and *B. caballi* (86.6%) supports the notion that while *B. ovis* shares a common ancestry with other *Babesia* species; it has diverged sufficiently to adapt to its specific ovine host.

The gene content and organization of the *B. ovis* mitochondrial genome are consistent with those observed in other apicomplexan parasites, encoding the three core protein-coding genes (*cox1*, *cox3*, and *cob*) and six large subunit (*LSU*) *rRNA* gene fragments. The conservation of these genes across *Babesia* and *Theileria* species underscores their essential role in mitochondrial function and highlights their potential utility as molecular markers for phylogenetic studies [19,22,25,36]. However, the absence of tRNA genes and other essential translation machinery in apicomplexan mitochondrial genomes, which are typically imported from the nuclear genome, remains a fascinating aspect of their biology [2,11]. This feature likely reflects a streamlined mitochondrial genome that relies heavily on the host cell machinery, a characteristic that might be associated with the intracellular parasitic lifestyle of these organisms.

The conservation of key mitochondrial genes such as *cox1*, *cox3*, and *cob* across various *Babesia* species highlights their fundamental roles in the mitochondrial electron transport chain and energy production. These genes are critical for the parasite’s survival and proliferation, as they encode essential components of the respiratory complexes [7]. The high degree of conservation suggests that these genes are under strong purifying selection to maintain their functional integrity. In contrast, the observed divergence in certain regions of these genes, particularly in *cox3* [7], may reflect adaptive changes that enable *B. ovis* to optimize its mitochondrial function in response to specific host environments or metabolic demands. This divergence could influence the efficiency of electron transport and ATP synthesis, potentially affecting the parasite’s growth rate and virulence.

Understanding the functional relevance of both conserved and divergent regions in these mitochondrial genes is crucial. The conserved regions can serve as reliable targets for the development of broad-range molecular diagnostics and potential therapeutic agents that could inhibit essential mitochondrial functions in the parasite [5,37,38]. Conversely, the divergent regions may offer opportunities to develop species-specific diagnostic tools and treatments, minimizing off-target effects on the host or non-target organisms. Additionally, these differences could provide insights into mechanisms of drug resistance, as mutations in mitochondrial genes have been associated with altered sensitivity to certain anti-parasitic drugs.

The discovery that the *B. ovis* mitochondrial genome is smaller than those of *B. microti*, *B. rodhaini* [7,8], and *T. equi* [7] but larger than that of *Toxoplasma gondii* [39] suggests a potential correlation between genome size and the parasite’s life cycle, host range, and pathogenicity. Smaller genomes might be indicative of a more specialized or simplified parasitic strategy, whereas larger genomes could reflect greater complexity in life cycle stages or a broader host range. Further comparative genomic studies could explore these hypotheses, shedding light on how mitochondrial genome size influences the biology and ecology of these parasites.

Incorporating mitochondrial genomic data, such as that from *B. ovis*, into broader phylogenetic studies could enhance our understanding of the evolutionary history and relationships within Piroplasmida. The combination of sequence data with structural genomic features may help resolve lingering ambiguities in the phylogenetic tree of this group, leading to more accurate classifications and a better understanding of the evolutionary processes that shape these important parasites [4]. The conservation of coding gene sequences and LSU rRNA fragments across *Babesia* and *Theileria* species, despite differences in TIR length and sequence, suggests that TIRs might play a more significant role in host adaptation and environmental resilience than previously thought [7]. The variations in TIRs could contribute to species-specific differences in how these parasites interact with their hosts, respond to environmental stresses, or establish infections. This aspect of apicomplexan biology warrants further investigation, particularly in the context of understanding how these parasites evolve and adapt to changing ecological conditions.

Overall, our findings underscore the importance of mitochondrial genome studies in unraveling the complexities of parasite biology and evolution. By highlighting both conserved and divergent genetic elements, we provide a foundation for future research aimed at developing novel diagnostic methods and therapeutic strategies. The insights gained from the mitochondrial genome of *B. ovis* not only enhance our understanding of its biology but also contribute to the broader knowledge of mitochondrial function and adaptation in apicomplexan parasites. This information is pivotal for devising effective control measures against babesiosis, improving animal health, and mitigating economic losses in the livestock industry.

## 5. Conclusions

In conclusion, this study provides a foundational understanding of the *B. ovis* mitochondrial genome, offering valuable insights into its genetic structure, evolutionary relationships, and potential functional adaptations. The data generated here not only contribute to the broader field of apicomplexan genomics but also lay the groundwork for future research aimed at developing better diagnostic and therapeutic tools for managing babesiosis in livestock. Continued exploration of mitochondrial genomics in *Babesia* species will likely reveal further complexities and novel targets for controlling these important parasites.

## Figures and Tables

**Table 1:** Primers used for amplifying the *B. ovis* mitochondrial genome.

Primer Name	Primer Sequence (5′–3′)
MitoF1	AACAAGTGATCATGTATAAAGTA
Mitoseqfor1	TGGGCTCATCATATGTACAC
Mitoseqrev1	GTGTACATATGATGAGCCCA
MitoF2	GCATGCAATACCGAACAGGGCCA
MitoR1	ACTCTATAGGTATTTGACGTAATT
Mitoseq2for	TTATTTCAAATCTATATAGT
Mitoseq3f	AGCCGATATAGAGTTTCA
MitoR2	TGTTCAACAGACGCTCCTCA
MitoF3	AACGACTTCTCTATTGTCTCCAC
Mitoseqrev2	TTCTTTGCCTTGGATGTCAAT
MitoR3	AATGAGTTATTGGGGAGC
MitoR5	TGTTAAAAAACTTTATATTTGTTGAAATTT

**Table 2:** Comparison of mitochondrial genome features among *Babesia* and *Theileria* species, including Terminal Inverted Repeats (TIRs) and gene lengths (bp).

Species	5′ TIR	cox1	cox3	LSU1	LSU3	LSU6	LSU2	cytb (cob)	LSU5	LSU4	3′ TIR	Total
*B. ovis* (PP973837)	117	1434	639	303	111	38	36	1092	68	82	117	6015
*B. bovis* (AB499088)	119	1434	639	302	111	38	35	1092	68	82	119	5970
*B. bigemina* (AB499085)	65	1434	639	299	111	37	36	1092	70	82	65	5924
*B. caballi* (AB499086)	62	1434	639	301	111	37	35	1092	68	82	62	5847
*B. gibsoni* (AB499087)	74	1434	639	306	111	43	35	1092	70	82	74	5865
BspXJ (KX698108)	25	1434	639	302	111	37	36	1092	69	82	25	6020
BmLT (KX698109)	35	1434	639	297	111	37	36	1092	70	82	35	5790
BmNX (MN605891)	101	1434	639	297	111	37	36	1092	70	82	101	5946
*T. parva* (AB499089)	94	1440	642	301	111	38	38	1092	68	82	94	5924
*T. orientalis* (AB499090)	47	1437	642	310	111	38	38	1092	69	82	47	5957
*T. luwenshuni* (MZ231018)	123	1458	636	299	111	38	37	1092	69	82	123	6000

## Data Availability

The raw data supporting the conclusions of this article will be made available by the authors upon request.

## Supplementary Materials

The following supporting information can be downloaded at https://www.mdpi.com/article/10.3390/vetsci11110554/s1. Figure S1: Alignment of mitochondrial genomes from various *Babesia* species, with primer binding sites highlighted in red. Conserved regions are marked with asterisks (*), indicating high sequence similarity across species.
