# Supplementary material for: Mitochondrial Genome Analysis of Babesia ovis (Apicomplexa: Babesiidae) Endemic in Sheep in Türkiye
Source: Vet Sci. 2024 Nov 10;11(11):554. doi: 10.3390/vetsci11110554 (PMC11598858; doi:10.3390/vetsci11110554)
Supplement: Supplementary file 1 [file vetsci-11-00554-s001.zip › vetsci-3236742-supplementary.pdf]

```
B.gibsoni-AB499087.1          ------------------------------AGCTTAATAAAA-----------------
B.bigemina-AB499085.1         -----------------------------------------------------------
B.caballi-AB499086.1          -----------------------------------------------------------
B.orientalis-KF218819.1       ------TGTTAAAAAACTTTATATTTGTTGAAATTTAAACAAGTGATCATGTATAAAGTA
B.bovis-AB499088.1            AAAAAAGTGTTAAAAACTTTATACATTAAAAAATTTAAACAAGTGATCATGTATAAAGTA
                                                                           mitoF1
B.gibsoni-AB499087.1          -------------------------------------GAGAATTTTTATTAGTTTTAT
B.bigemina-AB499085.1         -------------------------------------CAGCTTTGCTTTGAG----TTG
B.caballi-AB499086.1          -------------------------------------CAACACATTTGAAAG----TAT
B.orientalis-KF218819.1       CACTTGTTACAGT-TAAATAACAAAAACTGATGAATTTCAAATTTGTTGAAATTTTCTGT
B.bovis-AB499088.1            CACTTGTTACTGTGTAAATATCAAAAACAATTTAATTTCAAAATTTTTGAAA-----TAT
                                                                     *       *         *
B.gibsoni-AB499087.1          TAAGTTGTATTTTTTTAAAAAAAGTTTT------AAGTTGTTTATTGTTTATGCTTCAGA
B.bigemina-AB499085.1         TTTGTTAGTGTTTTTT-AATAAAGTTTTTTAAGAAAAGATATTA-TGTTTATGATTCAAA
B.caballi-AB499086.1          TTCTTTATTTTTTTGTAAAAAAAGTTTTTTTTGAAAATTA-----TATTTGTGATTCAAG
B.orientalis-KF218819.1       TTTTTTTGTTTATTGT--TAAAAGTTTTT-----AAGT--ATTATTATTTATGACATTAG
B.bovis-AB499088.1            GTTTTTTGTGTTGTGT-TATAAAGTTTTTTTTCAAAATTATATA-TGTTTGCATTTGCTG
                                  **    *  * *    ********      **         * ***
B.gibsoni-AB499087.1          GTTATAATTCAGTTTCAGCAAATCATAAAATTATAGGAATATCTTATATATGGTTAGCAT
B.bigemina-AB499085.1         GTTATAATTCGGTTTCAGCAAATCACAAAATTATAGGAATATCTTATATATGGTTATCAT
B.caballi-AB499086.1          GTTATAATTCAGTTTCAGCGAATCATAAGATTATAGGTATATCTTATATATGGTTATCAT
B.orientalis-KF218819.1       GTTATAATTCTGTTTCAGCAAATCACAAAATCATAGGAATATCCTATATATGGCTGTCTT
B.bovis-AB499088.1            GATATAGTTCGGTCTCTGCAAACCATAAAGTCATCGGTATATCCTACATATGGCTTTCAT
                              * **** *** ** ** ** ** ** **  * ** ** ***** ** ****** *  * *
B.gibsoni-AB499087.1          ATTGGTTTGGTGTGATAGGATTTTATATGAGTATATTGATAAGAACAGAATTAAGTATGA
B.bigemina-AB499085.1         ATTGGTTCGGTGTGATAGGATTCTATATGAGTATATTGATCAGAACTGAATTAAGCATGA
B.caballi-AB499086.1          ACTGGTTTGGTGTAATAGGATTCTATATGAGTATATTAATTAGAACCGAATTGAGTATGA
B.orientalis-KF218819.1       ATTGGTTCGGTGTTATTGGATTTTATATGAGTATATTAATTAGAACGGAATTAAGTATGA
B.bovis-AB499088.1            ATTGGTTTGGAGTTATTGGATTTTATATGAGTATTTTGATAAGAACAGAATTGAGTATGA
                              * ***** ** ** ** ***** *********** ** ** ***** ***** ** ****
B.gibsoni-AB499087.1          GTGGGTTGAAGTTAATTACTATGGATACTCTTGAAGTATATAACATGTTATTTTCTCTTC
B.bigemina-AB499085.1         GTGGATTAAAAATAATCACAATGGATACTTTAGAAGTATATAATATGTTGTTCTCTCTTC
B.caballi-AB499086.1          GTGGTATAAAGATATTAACTATGGATACTTTGGAGGTGTATAACATGTTGTTTTCTCTTC
B.orientalis-KF218819.1       GTGGATTGAAGATTATAACCATGGATACATTGGAGGTTTACAATATGTTGTTTTCTCTTC
B.bovis-AB499088.1            GTGGTTTAAAGATTATGACAATGGATACTCTTGAGATATACAATATGATGTTTTCTCTAC
                              ****  * **  *  * ** ********  * **  * ** ** *** * ** ***** *
B.gibsoni-AB499087.1          ATGGATTAATTATGATATTTTTTAATATTATGACTGGTTTATTTGGAGGTATTGGAAATT
B.bigemina-AB499085.1         ATGGTTTAATTATGATATTCTTTAACGTTATGACTGGTCTATTTGGTGGAGTTGGAAATT
B.caballi-AB499086.1          ATGGTTTAATTATGATATTTTTTAATATTATGACAGGATTATTTGGTGGAATTGGTAATT
B.orientalis-KF218819.1       ACGGTTTGATTATGATATTCTTTAATATTATGACTGGGCTTTTTGGAGGTATTGGAAATT
B.bovis-AB499088.1            ATGGTTTAATTATGATATTCTTTAATGTTATGACAGGTTTATTTGGTGGTATCGGAAATT
                              * ** ** *********** *****  ******* **  * ***** **  * ** ****
B.gibsoni-AB499087.1          ATTTATTTCCTATATTATTAGGTGCATGTGATGTTGTATTTCCTAGGGCAAATTTATATA
B.bigemina-AB499085.1         ATTTGTTTCCAATTGTTCTTGGAGCAGCTGATGTTGTTTTTCCAAGGGCAAACTTATACA
B.caballi-AB499086.1          ATTTATATCCAATAGTTCTTGGAGCTTGTGATGTTGTTTTTCCAAGGGCAAACTTATATA
B.orientalis-KF218819.1       ATTTATTTCCGATAATTCTTGGAGCTGCTGATGTTGTATTCCCTAGGGCAAATTTGTATA
B.bovis-AB499088.1            ATTTATATCCTATTATTTTAGGTGCAGCTGATGTTGTTTTTCCAAGAGCAAATTTATACA
                              **** * *** **  *  * ** **   ********* ** ** ** ***** ** ** *
B.gibsoni-AB499087.1          GTTTATTGCTTCAGCCAATAGCTTTCTGTTTGGTTATTGCTTGTATGTATGTTGAAATAG
B.bigemina-AB499085.1         GTTTGTTGCTTCAGCCAATTGCATTTGGTCTTGTAATTGCAAGTGTATATTTTGAAATGG
B.caballi-AB499086.1          GTTTGCTTCTTCAGCCAATTGCTTTTGGTCTTGTTGTAGCTTGTGTTTATTTGGAGATGG
B.orientalis-KF218819.1       GTTTGTTATTACAACCAATAGCTTTTGGTCTTGTTATAGCTTCTGTATATTTAGAAATGG
B.bovis-AB499088.1            GTTTGTTTTTACAGCCTGTTGCTTTTGGTCTTGTTATAGCTTCTGTATATTTGGAAATGG
                              ****  *  * ** **  * ** **  ** * **  * **   * * *** * ** ** *
B.gibsoni-AB499087.1          GAAGTGGTACTGGATGGACTTTATACCCTCCTTTGTCTACTTCTATATCTTCAGTAGGTA
B.bigemina-AB499085.1         GAAGTGGAACTGGATGGACATTGTACCCTCCTTTGTCAACTTCTATTTCTTCTCTTGGTG
B.caballi-AB499086.1          GAAGTGGTACTGGATGGACTTTGTATCCTCCTTTGTCAACTTCTATTTCCTCAATAGGTG
B.orientalis-KF218819.1       GAAGTGGTACTGGATGGACTTTATATCCTCCTTTATCTACTTCTGTTTCTTCAGTTGGAG
B.bovis-AB499088.1            GAAGTGGTACAGGTTGGACATTATATCCTCCTTTATCAACTTCAATATCTTCACTTGGTG
                              ******* ** ** ***** ** ** ******** ** *****  * ** **  * **
B.gibsoni-AB499087.1          TAGATTTTATAATATTTGGTTTACTTGCCTCAGGTATAGCTAGTGCTATGAGTGGTGCAA
B.bigemina-AB499085.1         TTGATTTTATTATATTTGGATTACTTGCTTCTGGAATTGCAAGTGCAATGAGTGGAGCTA
B.caballi-AB499086.1          TTGATTTTATAATATTTGGATTACTTGCTTCTGGTATTGCAAGTGCAATGAGTGGAGCTA
B.orientalis-KF218819.1       TTGACTTTATAATATTTGGTTTATTAGCTTCTGGAATAGCTAGTGCTATGAGTGGTGCAA
B.bovis-AB499088.1            TTGATTTTATAATATTTGGATTATTAGCTTCTGGAATTGCAAGTGCAATGAGTGGTGCTA
                              * ** ***** ******** *** * ** ** ** ** ** ***** ******** ** *
B.gibsoni-AB499087.1          ATTTTATAACTACTTTTGGCGCTTTAAAATCAATTGGTCAAACTGTAGATAGATTAATAC
B.bigemina-AB499085.1         ACTTTATAGTTACTTTTGGAGCTTTAAAATCTATTGGTCAAACAATAGACAGATTAGGTC
B.caballi-AB499086.1          ATTTTGTTACAACTTTTGGAGCTTTGAAGTCAATTGGTCAAACTATAGATAGATTGAATC
B.orientalis-KF218819.1       ATTTTGTAGTTACGTTTGGAGCTCTTAAATCAATTGGTCAAACAATCGATAGAATAAGCG
B.bovis-AB499088.1            ATTTTGTTGTTACTTTTGGAGCTTTAAAGTCAATTGGACAAACTATAGACAGATTAAGTG
                              * *** *    ** ***** *** * ** ** ***** *****  * ** *** *
B.gibsoni-AB499087.1          CTACAGTATGGTCAATAGTTTTAACTTCTTTTTTATTATTATTATCTCTTCCTGTTGTAA
B.bigemina-AB499085.1         CAATAGCTTGGTCAATTGTATTAACAGCATTCTTGTTGTTAATTTCTCTTCCAGTTGTTA
B.caballi-AB499086.1          CAGTTGCTTGGTCAATATTATTGACTTCTTTCTTGTTGTTGATTTCTCTTCCAGTTGTTA
B.orientalis-KF218819.1       CTGTCGCTTGGTCAATAGTATTGACTGCGTTTTTATTGTTAGTATCGTTACCTGTTGTTA
B.bovis-AB499088.1            TTTTAGCCTGGTCAATTGTATTAACTGCTTTCTTGCTTTTAGTTTCTCTTCCTGTTGTAA
                                   *  ********  * ** **  * ** **  * **  * **  * ** ***** *
B.gibsoni-AB499087.1          CATCAGTTTTATTGATGGTATTTATGGATAGACACTATAATACTATGTTTTTTGAATCTT
B.bigemina-AB499085.1         CTTCTGTTTTATTGATGGTATTCTTTGATAGACATTATAATACAATGTTCTTTGAGTCCT
B.caballi-AB499086.1          CTTCTGTTTTATTGATGGTATTTTTTGATAGACATTATAATACAATGTTTTTCGAATCTT
B.orientalis-KF218819.1       CATCAGTTTTGTTAATGGTTTTTCTTGATAGACATTATAATACAATGTTTTTTGAATCAT
B.bovis-AB499088.1            CTTCTGTTCTTTTAATGGTATTTTTAGATCGTCATTATAATACAATGTTCTTCGAATCTT
                              * ** *** * ** ***** **  * *** * ** ******** ***** ** ** ** *
B.gibsoni-AB499087.1          CAAATTCTGGAGATCCTATATTGTATCAGCATTTGTTTTGGTTTTTTGGTCATCCTGAAG
B.bigemina-AB499085.1         CAAATTCAGGAGATCCAGTATTATATCAACATTTGTTTTGGTTCTTTGGTCATCCAGAGG
B.caballi-AB499086.1          CAAATTCTGGAGATCCAGTGTTGTATCAGCATTTGTTTTGGTTCTTTGGACATCCTGAGG
B.orientalis-KF218819.1       CTAATGCTGGAGACCCAGTACTTTATCAACATTTGTTTTGGTTTTTTGGACATCCTGAGG
B.bovis-AB499088.1            CAAATTCCGGTGATCCAATTTTATATCAACATTTATTCTGGTTCTTTGGACATCCAGAAG
                              * *** * ** ** **  *  * ***** ***** ** ***** ***** ***** ** *
```

```
B.gibsoni-AB499087.1        TTTATATATTAATATTACCAGCTTTTGGAATAGTAAGTTTAATTTTATCTTGCTTTTGTT
B.bigemina-AB499085.1       TATATATTTTGATATTACCTGCTTTTGGTGTGATAAGTGCTATATTATCTTCTTATTGTA
B.caballi-AB499086.1        TTTATATTTTAATATTACCAGCATTTGGAATAGTAAGTTTAATATTATCTTGTTATTGTT
B.orientalis-KF218819.1     TTTATATACTTATATTACCTGCTTTTGGAATTGTAAGTTTGATATTATCATGTTACTGTT
B.bovis-AB499088.1          TATATATACTTATATTACCAGGATTTGGAATAATAAGTCTTATAGTTTGTACATATTGTG
                            * *****  * ******** *  *****  *  *****   **  * *     *  ***

B.gibsoni-AB499087.1        CAAAAGAAGTATTTGGAAATCAAACAATGATATTAGCTATGGTTTCTATAGCTTTATTAG
B.bigemina-AB499085.1       CAAAAGAGCTATTTGGAAGTCAAACTATGATATTAGCTATGATTTCAATCGCTTTACTTG
B.caballi-AB499086.1        CAAGAGAATTGTTTGGAAATCAAACAATGATTTTAGCGATGATTTCAATTGCATTATTAG
B.orientalis-KF218819.1     CCAAAGAATTGTTTGGAAATCAAACTATGGTTTTAGCCATGGCTGCTATAGCTTTGTTGG
B.bovis-AB499088.1          GAAAGGAATTATTTGGTAATTTAACAATGATATTAGCAATGATTTCTATAGCTTTATTAG
                              *  **  * ***** * *  *** *** * ***** ***  * * ** ** **  * *
                                        mitoseqfor1/rev1
B.gibsoni-AB499087.1        GATGTTTAGTATGGGCTCATCATATGTACACTTCTGGATTAGAAGCAGATACTAGAGCTT
B.bigemina-AB499085.1       GTTGTCTTGTATGGGCTCATCATATGTATACTTCAGGATTGGAGGCTGATACTCGTGCAT
B.caballi-AB499086.1        GATGTTTAGTTTGGGCTCATCATATGTATACTTCTGGATTGGAAGCAGATACTCGTGCTT
B.orientalis-KF218819.1     GTTGTTTAGTATGGGCTCATCATATGTACACATCAGGTTTAGAAGCAGATACACGTGCTT
B.bovis-AB499088.1          GTTGTTTGGTTTGGGCTCATCATATGTATACATCAGGTTTGGAAGCAGATACTCGTGCTT
                            * *** * ** **************** ** ** ** ** ** ** *****  * ** *

B.gibsoni-AB499087.1        TCTTCACAACAACTACAATATTAATTGCTTTACCAACAGGTAATAAAATATTTAATTGGG
B.bigemina-AB499085.1       TCTTTACAACCACTACAATATTAATTGCTTTACCAACTGGTAATAAAATATTTAACTGGG
B.caballi-AB499086.1        TCTTTACAACTACAACTATATTGATTGCATTACCTACAGGAAACAAAATATTCAATTGGG
B.orientalis-KF218819.1     TCTTTACGACAACTACAATATTGATAGCTTTACCAACTGGTAATAAAATATTTAATTGGG
B.bovis-AB499088.1          TCTTTACAACTACTACAATTTTAATTGCATTACCAACAGGTAATAAGATATTTAATTGGG
                            **** ** ** ** ** ** ** ** ** ** ***** ** ** ** ** ***** ** ****

B.gibsoni-AB499087.1        TATGTACTCTTCAGGGGTCAGAAAATATTAGAAGCATAGGAATAGTTATGCTTTCTATTT
B.bigemina-AB499085.1       TATGTACAGTACAAGGTGCTGAATCAGTAAGGAGTTTAGGAATAGTTATGATGGCTATTC
B.caballi-AB499086.1        TATGTACTCTTCAAGTTGTAGAAGGTGTTAAAAATTTAGGAGTTGTTTTAATGACTATAT
B.orientalis-KF218819.1     TTTGTACATTACAATGTGTTGAAGATATTAGAAATTTAGGAGTAGTTATGATGGCAATTT
B.bovis-AB499088.1          TATGTACTCTTCAAGAAGCAGTTGAAGTAAGAAATTTAGGAACAGTTTTGATGTCAATTA
                            * *****  * **        *       * *  *   *****   *** *  *  * **

B.gibsoni-AB499087.1        TATTTATAGTAAATTTTGTTATAGGTGGTACTACAGGTGTTATACTGGGAAATGCTGGAG
B.bigemina-AB499085.1       TATTTGTTGTAAACTTCGTTATTGGAGGTACTACTGGAGTCATACTCGGAAATGCTGGTG
B.caballi-AB499086.1        TATTTGTTGTTAATTTTGTTATTGGAGGTACAACTGGAGTTATACTTGGAAATGCAGGAG
B.orientalis-KF218819.1     TATTTGTAATAAACTTTGTTATAGGAGGTGTTACTGGAGTAATATTAGGTAATGCTGGAG
B.bovis-AB499088.1          TGTTTGTTATAACTTTTGTAATAGGAGGTGTTACAGGTGTCATACTTGGAAACGCTGGAG
                            * *** *  * *  ** ** ** ** ***   ** ** ** *** * ** ** ** ** *

B.gibsoni-AB499087.1        TAGATATTACTCTTCATGATACATTATATGTCGTTGGTCATTTCCACTTTGTTCTTTCAA
B.bigemina-AB499085.1       TTGATGTTTCCTTACACGATACTTTGTATGTTGTTGGACACTTCCACTTTGTTCTATCAA
B.caballi-AB499086.1        TTGATGTTTCTCTACACGATACTTTGTACGTTGTAGGTCATTTCCACTTTGTATTGTCAA
B.orientalis-KF218819.1     TTGACATATCTTTACATGATACTTTGTATGTTGTTGGTCATTTCCATTTTGTACTTTCAA
B.bovis-AB499088.1          TAGATATATCTTTGCATGATACTTTATATGTTGTAGGTCATTTTCACTTTGTTTTATCAA
                            * **  *  *  * ** ***** ** ** ** ** ** ** ** ** *****  * ****

B.gibsoni-AB499087.1        TTGGAGCTATTATAGGACTGTTATGTTTTATAATATTTGCTCAAAGATTAATAATGGGTA
B.bigemina-AB499085.1       TTGGAGCTATAATTGGTTTATTGTGCTTTATATTCTTTATCCAAAGATTATTAGTAGGTA
B.caballi-AB499086.1        TTGGAGCTATAATGGGTTTATTGTGCTTTATATTCTTTATTCAAAGAATGTTAATAGGTA
B.orientalis-KF218819.1     TTGGAGCTATAATTGGTTTATTATGCTTTATATTTTATGTTCAAAGATTGCTAGTAGGTA
B.bovis-AB499088.1          TTGGAGCTATAATAAGTTTATTGAGTTTCATATTCTATTGTCAAAGACTTCTAGTTGGAA
                            ********** **  *  * **  * ** *** * * *   ****** *  ** * ** *

B.gibsoni-AB499087.1        CTATCTTTTCAAATAAAACAGTATTATTTATAATCCCAATATTTATGAGTGCTGTATTCT
B.bigemina-AB499085.1       CAATATTCACTAATCAAATGATACTCTTTATAATTCCAATATTTATGATATCAGTTTTCT
B.caballi-AB499086.1        CAATTTTTTCAAATCAAATGATACTTTTTATAATTCCAATATTTATGAGTTCAGTGTTCC
B.orientalis-KF218819.1     CTATATTCTCAAATCAAATGATATTACTTATAATACCTACGTTTATGTCTTCTGTATTCT
B.bovis-AB499088.1          CAATATTCTCTAATCAATTAATACTTATTATAATACCTGCTTTTATGGCTGGAATATTCT
                            * ** **  * *** **    ** *  ******* **    ******        * ***

B.gibsoni-AB499087.1        TAACATTTATACCAATGCACTTTCTTGGATTTACTCCATTACCAAGGAGAATACCAGATT
B.bigemina-AB499085.1       TAACATTCTTACCAATGCATTGTCTTGGATTCAGTCCTTTACCAAGGAGAATACCTGATT
B.caballi-AB499086.1        TTACATTCCTTCCAATGCATTTCTTAGGATTTAGTCCTTTACCAAGGAGAATTCCTGATT
B.orientalis-KF218819.1     TAACTTTCTTACCAATGCATTTTTTGGGATTCAGTCCTTTACCAAGGAGAATACCAGATT
B.bovis-AB499088.1          TAACTTTTATGCCAATGCATTTCTTAGGATTTACTCCTCTTCCAAGGAGGATACCTGATT
                            * ** **  * ******** *   * ***** * ***  * ******** ** ** ****

B.gibsoni-AB499087.1        ATGCAGATGAAATGTGGGGGTGGAATTTTCTATGTACTATTGGTTCAACTATGATGTTAA
B.bigemina-AB499085.1       ATCCTGACGACATGTGGGGATGGAATTTCTTATGTACTATTGGATCTACTATGATATTGA
B.caballi-AB499086.1        ATGCTGATGAAATGTGGGGATGGAATTTTCTATGTACAATCGGATCTACTATGATATTAA
B.orientalis-KF218819.1     ATCCTGATGAAATGTGGGGATGGAATTTCTTATGTACAATAGGTTCAACTATGATATTTG
B.bovis-AB499088.1          ATCCTGATGATATGTGGGGATGGAATTTCTTGTGTACTATTGGTTCAACAATGATATTCT
                            ** * ** ** ******** ********  * ***** ** ** ** ** ***** **
                                                                                   mitoF2
B.gibsoni-AB499087.1        TATTAAAATTAATAATATTATTATTCATATCTTTAT-AAAAATACACAAGGCATGCAATA
B.bigemina-AB499085.1       TATTAAAATTAATCATAGTAATATTTATTTCATTATAAAAAAATACACAAGGCATGCAATA
B.caballi-AB499086.1        TTTTAAAAATAATTATAGTACTATTTATAGCATTAT-AAAAATACACAAGGCATGCAATA
B.orientalis-KF218819.1     TGTTGAAGTTTATTATTGTACTATTTATTTCATTATAAAAAGTACACAAGGCATGCAATA
B.bovis-AB499088.1          TCTTAAAGTTAATTACTGTAATATTCATATCATTATAAAAATTACACAAGGCATGCAATA
                            * ** **  * ** *   ** **** **  * **** **** *****************

B.gibsoni-AB499087.1        CCGAACAGGGCCATATATATA-----TATTTCATTTTTTGCATCACTGCTAA--TATTTA
B.bigemina-AB499085.1       CCGAACAGGGCCATTTATTTTTATATTATTTCATTTTTTGCATCACTGCTAT-ATCTTTA
B.caballi-AB499086.1        CCGAACAGGGCCATTTATTTT--CATTTTTCATTTTTTGCATCACTGCTAACTTTTTA
B.orientalis-KF218819.1     CCGAACAGGGCCACTTATTTT---------TCATTTTTTGCATCACTGCTAAATATTTA
B.bovis-AB499088.1          CCGAACAGGGCCATTTATTTT---------TCATTTTTTGCATCACTGCTAA-TTTTTA
                            *************  *** *         ********************     ****

B.gibsoni-AB499087.1        GCTAAAAACAGAAC--TTTAAACCATAACTTCAACTTATTAATATATTTTTCTTGTTAAC
B.bigemina-AB499085.1       GCT-AAAACAGAACTTTTTAAACCATAACTTCAACTTATTAATATATTTTTCTTGTTAAC
B.caballi-AB499086.1        GCT-AAAACAGAAC-TTTTAAACCATAACTTCAACTTATTAATATATTTTTCTTGTTAAC
B.orientalis-KF218819.1     GCT-AAAACAGAAC-TTTTAAACCATAACTTCAACTTATTAAAATATTTTTCTTGTTAAC
B.bovis-AB499088.1          GCT-AAAACAGAAC-TTTTAAACCATAACTTCAACTTATTAATATATTTTTCTTGTTAAC
                            *** **********  ************************** *****************
```

```
                                                    mitoR1
B.gibsoni-AB499087.1          TATTTGTACGATAATTACGTCAAATACCTATAGAGTCATCAGTGATTTAATCCTTTATAT
B.bigemina-AB499085.1         TATTTGTACGATAATTACGTCAAATACCTATAGAGTCATCACTGATTTAATCCTTGTTAT
B.caballi-AB499086.1          TATTTGTACGATAATTACGTCAAATACCTATAGAGTCATCAGTGATTTAATCCTTGTTAT
B.orientalis-KF218819.1       TATTTGTACGATAATTACGTCAAATACCTATAGAGTCATCATTGATTTAATCCTTGTTAT
B.bovis-AB499088.1            TATTTGTACGATAATTACGTCAAATACCTATAGAGTCATCGATGATTTAATCCTTGTTAT
                              ****************************************  *************  ***

B.gibsoni-AB499087.1          TTTTAATGGGACCATGAGTTTATATGTACCATTTTAGTAATTTAAAAAAGATATCATAAG
B.bigemina-AB499085.1         TTTTAATGGGACCATGAATTTATATGTACCATTCTAGTAATTTAAAAAAGAATTCATTAG
B.caballi-AB499086.1          TTTTAATGGGACCATGAGTTTATATGTACCATTTTAGTAATTTAAAAAAGACCTCATAAG
B.orientalis-KF218819.1       TTTTAATGGGACCATGAGTTTATATGTACCATTTTAGTAATTTAAAAAAGAATTCATAAG
B.bovis-AB499088.1            TTTTAATGGGACCATGAGTTTATATGTACCATTTTAGTAATTTAAAAAAGAATTCATTAG
                              ***************** *************** *****************  **** **

B.gibsoni-AB499087.1          TAACCTCTGTGAAAGTG----AAATAGCGAGATGGGAATTGTGCTTCTATAGC-------
B.bigemina-AB499085.1         TAACCTTTGTGAAAGTGAAAAAAATAGCGAGATGGGAATTGTGCTTCTATAGAAAAAA--
B.caballi-AB499086.1          TAACCTCTGTGAAAGTG----AAATAGCGAGATGGGAATTGTGCTTCTATAGAAAAAAAA
B.orientalis-KF218819.1       TAACCTCTGTGAAAGTG----AAATAGCGAGATGGGAATTGTGCTTCTATAGAAAAA---
B.bovis-AB499088.1            TAACCTCTGTGAAAGTG----AAATAGCGAGATGGGAATTGTGCTTCTATAGAAAAC---
                              ****** **********    *******************************

B.gibsoni-AB499087.1          -TTTTTATAATATATTTTGAATCTCGAAAAAGTAGAAAAAAGATAGCGTTATAGCTCTTA
B.bigemina-AB499085.1         -TTTGTATAATATTTTTTGAATCTCGAAAAAGTAGAATAAAGATAGCGTTATAGCTCTTG
B.caballi-AB499086.1          TTTTATGTAATATTTTTTGAATCTCGAAAAAGTAGAACAGAGATAGCGTTATAGCTCTTG
B.orientalis-KF218819.1       -TTTATATAATAT-TTTTGAATCTCGAAAATGTAGAATGAAGATAGCGTCATAGCTCTTG
B.bovis-AB499088.1            -TTAATATAATATTTTTTGAATCTCGAAAAAGTAGAATGAAGATAGCGTCACAGCTCTTG
                               **  * ****** **************** ******   ********* * *******

B.gibsoni-AB499087.1          CAGTCAGTACGAAGTCGAAACAAGGTAGTTGACAGTGAACTTGTAGCTGAACAAAGTAAA
B.bigemina-AB499085.1         CAGTCAGTACGAAGTCGAAACATGGTAGTTGACAGTGAACTTGCAGCTGAACAAAGTAAA
B.caballi-AB499086.1          CAGTCAGTACGAAGTCGAAACATGGTAGCTGACAGTGAACTTGCAGCTGAACAAAGTAAA
B.orientalis-KF218819.1       CAGTCAGTACGAAGTCGAAACATGGTAGCTGACAGTGAACTTGCAGCTGAAAAAAGTAAA
B.bovis-AB499088.1            CAGTCAGTACGAAGTCGAAACATGGTAGCTGACAGTGAACTTGCAGCTGAATAAAGTAAA
                              ********************** ***** ************** ******* ********

B.gibsoni-AB499087.1          GAAACAGATGTAAAGTCAATAGCTTT-TAAAATAATTATGTTAAATTTCGATTACAATAA
B.bigemina-AB499085.1         GAAACAGATGTAAAGTCAATAGCTATATAATATAATTATGTTAAA-TTCGATTACAATAA
B.caballi-AB499086.1          GAAACAGATGTAAAGTCAATAGCTGT-TAATATAATTATGTTAAA-TTCGATTACAATAA
B.orientalis-KF218819.1       GAAACAGATGTAAAGTCAATAGCTTT-TAACTTAGTTATGTTATA-TTCGATTAAATT--
B.bovis-AB499088.1            GAAACAGATGCAAAGTCAACAGTTTT-TAATATAATTATATGAAA-TTCGATTAAGTT--
                              ********** ******** ** * * ***  ** **** * * * ********   *

B.gibsoni-AB499087.1          AGTTTCGTCC---TTTTTTAAACAAAGATGATATCCTTTTGTTGA-CTAAGTATAAGATT
B.bigemina-AB499085.1         AGTTTCGTCCTTTGTTTCTAAGCATTGATGATACCTTTTTGTTGATTTTAATAT---GTT
B.caballi-AB499086.1          AGTTTCGTCC-TTGTTTCTTAGCATTGATGATACCTTTTTGTTGATTTGTATAT---GTT
B.orientalis-KF218819.1       ATTTTCGTCC-----TCTTTAACAAAGATGATACCTTTTTGTTGATTTTTGTAT---GTT
B.bovis-AB499088.1            ATTTTCGTCC-----TTCTTAACATAGATGATACCTTTTTGTTGA--TTAATAT---GTT
                              * ********     *  * * **  ******* * *********  *   ***    **

B.gibsoni-AB499087.1          CAACAAGCATACGTATATAACATTGACGGTTATAAATACGTGTCGAGCAGTGTGTTCAAA
B.bigemina-AB499085.1         CAACAAGCATAAGTATATAACATTGAAGGTTATAGATACGTGCCGAGCAGTGTGTTCAAA
B.caballi-AB499086.1          CAACAAGCATAAATATATAACATTGAAGGTTATACATACGTGCCGAGCAGTGTGTTCAAA
B.orientalis-KF218819.1       CAACAAGCATAAATATATAACATTGAAGGTTATATATACGTGCCGAGCAGTGTGTTCAAA
B.bovis-AB499088.1            CAACAAGCATAAGTATATAACATTGAAGGTTATATATACGTGCCGAGCAGTGTGTTCAAA
                              ***********  ************* ******* ******* *****************

B.gibsoni-AB499087.1          GTGGTTATTACGCGAATTC-TTAAATTATAAAGATCAAGATGAATCCGATT---TGATAA
B.bigemina-AB499085.1         GTGGTTATTACGCGAATTCTTTAAATCATTAAGTTCAAGATGAATCCGATTAGATGTTAT
B.caballi-AB499086.1          GTGGTTATTACGCGAATTCTTTAAATTGTTAATTTCAAGATGAATCCGATTAGATGATAT
B.orientalis-KF218819.1       GTGGTTATTACGCGTAGTATTTAAATTGTTAAATTCAAGATGAATTCGATTAAATGTTAT
B.bovis-AB499088.1            GTGGTTATTACGCGTTTTCTTTAAATCATTAAGTTCAAGATGAATCCGGTTAGATGTTAT
                              **************   *  ******  * **  *********** ** **   ** **

B.gibsoni-AB499087.1          TATTATAAAAAAGGCTAATGATATTGTTTAATATCAGCATGGGATTATAAAACAGTATAT
B.bigemina-AB499085.1         TGTTATAAAAAAGGCTAATGATATCGTTTAATATCAGCATGGGATTATAAAACAGTATAT
B.caballi-AB499086.1          TGTTATAAAAAAGGCTAATGATATTGTTTAATATCAGCATGGGATTATAAAACAGTATAT
B.orientalis-KF218819.1       TGTTATAAAAAAGGCTAATGATATAATTTAATATCAGCATGGGATTATAAAACAGTATAT
B.bovis-AB499088.1            TGTTATAAAAAAGGCTAATGATATAATTTAATATCAGCATGGGATTATAAAACAGTATAT
                              * **********************  **********************************

B.gibsoni-AB499087.1          TATGTAAT-TATTTACTGTCAGCTAAAACGTATCAATGTCTCTACTCTGTTACCTTGAA-
B.bigemina-AB499085.1         TGAGTATTATATTTACTGTCAGCTAAAACTTACCAATATCTCTACTCTGTTACCTTGAAT
B.caballi-AB499086.1          TGAGTATTATATTTACTGTCAGCTAAAACGTATCAATGTCTCTACTCTGTTACCTTGAAT
B.orientalis-KF218819.1       TAAATAATATGGTTACTGTCAGCTAAAACGTATCAATGTCTCTACTCTGTTACCTTAAAT
B.bovis-AB499088.1            TGAGTATTGTGTTTACTGTCAGCTAGAACGTATCAATGTCTCTACTCTGTTACCTTGAAT
                              *   ** * *  ************* *** ** **** ****************** **
                                           mitoseq2for
B.gibsoni-AB499087.1          AAATATAATTATTTCAAATCTATATAGTATATGCATTTTCTTAACTTACATTAAGAAAAG
B.bigemina-AB499085.1         AAATATTATTATTTCAAATCTATATAGTACCTGTA--TTCTTAAATCACATTAAGAAAAG
B.caballi-AB499086.1          AAATTTTATTATTTCAAATCTATATAGTGTCCTTA-TTTCTTAAATTACATTAAGAATAA
B.orientalis-KF218819.1       AAATATTATTATTTCAAATCTATATAGTACTA----TTTCCTAATTTACATTAGGAAAAG
B.bovis-AB499088.1            GAATATAGTTATTTCAAATCTATATAGTACCG----TTTCCTAACTTACATTAGGAAAAG
                               *** *  ********************         *** *** * ****** *** *

B.gibsoni-AB499087.1          TAATAAAGTTAGAAAAACCCAAATAAACTCAACAAAATGCCAATATGTACCTAAACTAAT
B.bigemina-AB499085.1         TAGGAAAGTCAAAAATACCCAAACTAATTCAACAAAATGCCAGTAGGTTCCAATGTTAAA
B.caballi-AB499086.1          TAGGAAAGTTAAAGCTACCCAGATAAGTTCAACAAAATGCCAATATGTTCCAATATTGGT
B.orientalis-KF218819.1       GAGTAGTGTTAACACTACCCAGATTAATTCGACAAAATGCCAATAAGTTCCAATGTTGAA
B.bovis-AB499088.1            GAGCATTGTTAAAGCTACCCAGATTAATTCAACAAAATGCCAATATGTACCAATGTTAAA
                               *  *  ** *     ***** *  *  ** *********** ** ** ** *   *

B.gibsoni-AB499087.1          ATCTTCTACTCTATTTGATCCTCTATTTTCATGATAAAATATTGATGTTAATACTAATAC
B.bigemina-AB499085.1         ATCCTCAGATCTGTTTGAACCATCATTATCGTGATAATATATTGACAATAATATTAATAC
B.caballi-AB499086.1          ATCTTCTGCTCTATTTGAACCGTCATTTTCATGATAGTAAATAGATAGTACTATCAATAC
B.orientalis-KF218819.1       ATCTTCTACTTTGTTAGAACCATCATCTTCGTGATAATATATTGATAATAAAATAAATAC
B.bovis-AB499088.1            ATCTTCTGCTCTATTTGAACCATCACTATCGTGATAGTTAACTGATAAACATATAAATAA
                              *** **   * * ** ** **   *   ** *****    *  **       *  ****

B.gibsoni-AB499087.1          AGCACCTATACATACATGTGATGAATGTAAACCGGTGATTATTAAAAATAAAGTTGTAAA
B.bigemina-AB499085.1         TACTCCAACACATACGTGCATAGAATGTAGTCCTGTAATAATCAAGAAAGTAGTTATAAA
B.caballi-AB499086.1          AATTCCAACACAAACATGCAAGGAATGTAATCCTGTTATTATTAAGAAAGCAGTTACAAA
B.orientalis-KF218819.1       AGCTCCTACACAAACATGTAGTGAATGTAGTCCTGTAATAATAAAGAAGGCAATTGAAAT
B.bovis-AB499088.1            AACTCCTACACAAACGTGTAGTGAATGTAGTCCAGTTATAATTAAGAAAGCAAGAGAAAA
                                  ** * *** ** **    *******  ** ** ** ** ** **   *     **
```

```
B.gibsoni-AB499087.1          CCAACTGTGATTAACAGCTGATTGTAATAATGAATACTCATCACCTTGATAAGATATGAA
B.bigemina-AB499085.1         CCAACTGTGATTTAATCCACATTGTATAAAGGTGTACTCATCACCTTGGTATGATAAGAA
B.caballi-AB499086.1          CCAGTTATGATTTAATCCAGTCTGCAATAAAGAATATTCGTCTCCTTGATAAGATAAAAA
B.orientalis-KF218819.1       CCAACTGTAATTTAAACCACATTGTAAAAGACTATATTCATCGCTTTGATAAGATAAAAA
B.bovis-AB499088.1            CCAACTGTCGTTTAATCCGCATTGTAAAAATGTATATTCATCTCCTTGATAGGATAGGAA
                              ***  * *  ** *   *    ** *  *     ** ** ** * *** ** ****  **

B.gibsoni-AB499087.1          AGCTATAGCTACAAATGTTAATACAGCAACTAATTTTTCAGTTTCAGAATTTTCTCCTGA
B.bigemina-AB499085.1         TGCTACAGCTATTAAAGTAGCTATACTTAAAAGTTTCTCTGACTCAGAGAATTCTCCGGA
B.caballi-AB499086.1          TGCTATTGCTACTAAAGCTGACATACCTATAAATTTTTCAGCATCTGAATTTTCGGATGT
B.orientalis-KF218819.1       CAAAAAAGCAATAAATAAACATGTAATAAGCCATTTTTCAATATCAGAAAAATGTGAAAC
B.bovis-AB499088.1            TAATGAACCAATAAAGAATGCCGATGTCAACCATTTTTCAAAATCAGAAAAATGTGAAAC
                                      * *  **             *    *** **    ** **    *

B.gibsoni-AB499087.1          GATATTTTTATGAAGCATAAATGATACTATAACACTACCAATATTTAATGTACTAGTAAT
B.bigemina-AB499085.1         AGTATTTTTATAAATTATAAAAGATATAATACTACTAGCAACGTTTAAAGTACCAGTAAT
B.caballi-AB499086.1          AGCATTTTTATGCATTATGAAAGATATTATACTACTTGCAGCATTTAATGTACTAGTAAT
B.orientalis-KF218819.1       AGTTAATTTATAAACAAAAATAGATACTATACTACTAGTTATGTTCAATGAACTAGTTAT
B.bovis-AB499088.1            AGTCATTTTATACACTAGTATTGATATTACACTACTAGTTATATTTAAAGAACTAGTTAT
                                    *****  *  *  *  ****  * *  ***       ** ** * ** *** **

B.gibsoni-AB499087.1          ATTTAAAAATGGTTCAATATTAGAAGGTGTTTCTATAGAAACATTTCCCCATCTCATATG
B.bigemina-AB499085.1         GTAAAAGAAAGGAACCAAGTTAAGAGGAGTATCCATTGCGACGTTTCCCCATCTTACATG
B.caballi-AB499086.1          ATACATAAAAGGAACTAGATTTAAAGGAGTATCAATTAAAATATTTCCCCATCTTGCATG
B.orientalis-KF218819.1       ATTAGAATAAGGACCAATATTAACAGGTAAATCGTTACTGACACCACCCCATCTAAGATG
B.bovis-AB499088.1            ATTTAAATAAGGTTCAAGATTTAAAGGCATATCAATATATATGTTACCCCATCTTGCATG
                               *      * **  * *  **   ***    **  *    *     ********   ***

B.gibsoni-AB499087.1          TAAAAAGGCCCAAATGAACCCGAAAAATAACATTATTTCTGAAAATATAATCATACCATA
B.bigemina-AB499085.1         AAAATAAGCCCATGCAAATCCAAAGAATATTAATATTTCTGAAAAAATAATCATTCCAAA
B.caballi-AB499086.1          AAGGAATGCCCATATAAATCCAGAGAATAATAGTATTTCTGAAAATATAATCATACCAAA
B.orientalis-KF218819.1       AAAGTAAGCCCATATAAAACTGAAAAATAATAGTATTTCTGAAAATATAAGCATTCCGAA
B.bovis-AB499088.1            TAGATAATCCCATATGAAACCAAAAAATAGTAATATTTCTGAAAATATTAACATTCCAAA
                               *   *  ****    ** *   * ****  * ************ ** * *** **  *

B.gibsoni-AB499087.1          TATTGCAAATAAAGTAGATGTATTTGTGGTTATAAATTCTCTAATACTATTTACAATTGC
B.bigemina-AB499085.1         CACTGCATACAAAGTTGATGTATCAGTTGTCATAAATTCTCTAATACTATTAACTAAAGA
B.caballi-AB499086.1          TACTGCGTACAAAGTTGATGTATCAGTCGACATAAATTCTCTTATACTGTTAATTAAAGA
B.orientalis-KF218819.1       AACTGCATATAGAGTTGATGTATCAGTTGTAGAAAACTCTCTAATACTATTAACTGAAGA
B.bovis-AB499088.1            CACTGAATAAAGAGTTGAACTTTCTGTTGTAGAGAATTCTCTACTTGTATTAACTGAAGA
                               * **   * * *** **  * *  ** *     ** *****  *  * ** *     *

B.gibsoni-AB499087.1          TAAAAATGAAAACATAAATACTTCTCCTAAAGATGCTGCTTTTAATGAAGTAGCATATAA
B.bigemina-AB499085.1         TATTAAAGAAACCAAAAAGACTTCTCCCAAAGATGCACTTTGTAATGAAGTTGAAAACAA
B.caballi-AB499086.1          TACAAAAGAAACAAAAAATACTTCTCCAATAGATGCACTTTGTAGTGAAGTAGCGTATAG
B.orientalis-KF218819.1       CAAAAAAGATGTTAAAAATACTTCTCCCAAAGATACGCTTTGTAACGAAGTTGCAAATAA
B.bovis-AB499088.1            TACAAATGACAATAAAAATACTTCTCCCAAAGATACACTTTGTAGTGAAGTTGCAAATAA
                               *  ** **    * *** ******** * **** *   ** **  ***** *   * *

B.gibsoni-AB499087.1          ACTATTATGTTCTATATATGTTATAATGAAA---TTCTTATAAATGGAATCAGAATATTC
B.bigemina-AB499085.1         AGTTTGATTTTTTAGATAAATTATAAAGAAAAGGTTCTTATACATGGAATCAGAATATTC
B.caballi-AB499086.1          ACTTTGATTTTTCAAATAAATTATAAAGAAA-AATTCTTATACATGGAATCAGAATATCC
B.orientalis-KF218819.1       GCAATGGTTTTTTAAATATATTATAAAGAAA-AATTCTTATGTATGGAATCAGAATATTC
B.bovis-AB499088.1            GCTATGGTTCTTTAGATAAATTATAAAGAAACAATTCTTATGTATGGAATC-GAATATCC
                                  *  *  *  * ***  ****** ****   *******  ******** ****** *

                                                       mitoseq3f
B.gibsoni-AB499087.1          CAGGGTATCTAATCCTGTACTATAGAGCCGATATAGAGTTTCAAATAACGTTAGTTATAT
B.bigemina-AB499085.1         CAGGGTATCTAATCCTGTACTATAAAGCCGATATAGAGTTTCAAAT-ATGTTAATTATAT
B.caballi-AB499086.1          CAGGGTATCTAATCCTGTGCTATTAAGCCGATATAGAGTTTCAAAT-AAGTTAATTACAT
B.orientalis-KF218819.1       CAGGGTATCTAATCCTGTGCTATATAGCCGATATAGAGTTTCAAAT-ATGTCAATTATAT
B.bovis-AB499088.1            CAGGGTATCTAATCCTGTGCTATAAAGCCGATATAGAGTTTCAAAT-GTGTTAATTATAT
                              ****************** ****  *********************   ** * *** **

B.gibsoni-AB499087.1          TA--ATGTACTTTTAATATGATTGTACGCTGTTTCGTATTAGACTATATAGGTTAACTTA
B.bigemina-AB499085.1         TAATATGTACTTTAATTATGATTGTACGCTGTTTCGTATTAGACTATATAGGTTAACTTA
B.caballi-AB499086.1          TA--ATGTACTTTAAGTATGATTGTACGCTGTTTCGTATTAGACTATATAGGTTAACTTA
B.orientalis-KF218819.1       TA--ATGTACTTTAAATATGATTGTACGCTGTTTCGTATTAGACTATATAGGTTAACTTA
B.bovis-AB499088.1            TA--ATGTACTTTAAATATGATTGTACGCTGTTTCGTATTAGACTATATAGGTTAACTTA
                              **  ********* * ********************************************

                                                                      mitoR2
B.gibsoni-AB499087.1          CATAGTCCATGTATATATTAAATTAAGTAA--AATGAGGAGCGTCTGTTGAACAACTACA
B.bigemina-AB499085.1         CATAGTCCAA-------TTTATTTAAATAAAGAATGAGGAGCGTCTGTTGAACAACTACA
B.caballi-AB499086.1          CATAGTCCAC-------TTTATTTAAATAA--ACTGAGGAGCGTCTGTTGAACAACTACA
B.orientalis-KF218819.1       CATAGTCCAT-------TTTTATTAAAACA--AATGAGGAGCGTCTGTTGAACAACTACA
B.bovis-AB499088.1            CATAGTCCAT-------TTTATTTAAAACA--ATTGAGGAGCGTCTGTTGAACAACTACA
                              *********        **   ****   *  * **************************

B.gibsoni-AB499087.1          CAAGACAATGCTAAGATATATATCTATGTATATTTTCTGATACCTGTTCAGTTACAATTC
B.bigemina-AB499085.1         TAAGACAATGCTAAGATATATATCGAAGTACATTTTCTGACGCCTGTTCGGTGGTACTCT
B.caballi-AB499086.1          TAAGATAATGCTAAGATATATATCGAAGTATATTCTCTGATACCTGTTCAGTGGTATTTT
B.orientalis-KF218819.1       TAAGACAATGCTAAGATTTATATCTAAGTACATTTTCTGATGCCTGTTAATTAATATCTA
B.bovis-AB499088.1            TAAGACAATGCTAAGATTTTTATCAAAGTATATTTTCTGATACCTGTTCAGTGATATTTA
                               **** *********** * **** * *** *** *****  ******   *   *

B.gibsoni-AB499087.1          TATATTGTAAAACAACGGGAATTCCCTTAAAGTAGGAATTAATTGTCTAAATAACAATTA
B.bigemina-AB499085.1         ----GTATCAAACAACGGGGATTCCCTTAAAGTAGGAATTAATTGTCTAAATAACAATTA
B.caballi-AB499086.1          TTAAATACCTAACAACGGGAATTCCCTTAAAGTAGGAATTAACTATCTAAATAATGGTTA
B.orientalis-KF218819.1       ACAGATATTTAACAACTGGGATTCCATCAAAGTAGTAATTAATTGTCTAAATAACAATTA
B.bovis-AB499088.1            ACAAATATCCTACAACTGGGTTTCCATTAAAGTAGTAATTAATTGTCTAAATAACAATTA
                                   *     ***** **  **** * ******* ****** * *********   ***

                                                 mitoF3
B.gibsoni-AB499087.1          TGTGAATGGTATAACGACTTCTCTATTGTCTCCACTAAGTTCTCTATGAAATAGTCTTAG
B.bigemina-AB499085.1         TGTGAATGGCGTAACGACTTCTCTATTGTCTCCACTAAATCCTTTATGAAATAGTCGTAG
B.caballi-AB499086.1          TGAGAATGGTATAACGACTTCTCTATTGTCTCCACTAAATCCTTTTTGAAATAGTCGTAG
B.orientalis-KF218819.1       TGCAAATGGTACAACGACTTCTCTATTGTCTCCACTAAATTCTTTTTGAAATAGTCTTGG
B.bovis-AB499088.1            TGCAAACGGTAAAACGACTTCTCTATTGTCTCCACTAAGTCCTTTTTGAAATAGTCTTAG
                              **  ** **   ************************** * ** * ********** * *
```

```
B.gibsoni-AB499087.1         ATTTGAATAGAAATCTTGATATACTGGGACGGATAGACCCCGTGCACCTTTACTTCTCTT
B.bigemina-AB499085.1        ATTTGAATAGAAATCTTGGTATACTGTGACGGAGAGACCCCGTGCACCTTTACTTCTCTT
B.caballi-AB499086.1         ATTTGAATAGAAATCTTGATATACTGTGACGGAGAGACCCCGTGCACCTTTACTTCTCTT
B.orientalis-KF218819.1      ATTTGAATAGAAATCTTGATATACTGTGACGGAGAGACCCCGTGCACCTTTACTCCTCTT
B.bovis-AB499088.1           ATTTGAATAGAAATCTTGATATACTGTGACGGAGAGACCCCGTGCACCTTTACTTCTCTT
                             ****************** ******* ****** ******************** *****

B.gibsoni-AB499087.1         ---AAAAGTTTTGCAAAACTTTAAGGTATGGTGAGACGACATGGAGGTGTCAATAAATTA
B.bigemina-AB499085.1        -AAACAAGTTTTGCAAAACTCAAAGGTATGGTGAGACGACATGGAGGTGTCAATAAATTA
B.caballi-AB499086.1         -AAATAAGTTTTGCAAAACTTTAAGGTATGGTGAGACGACATGGAGGTGTCAATAAATTA
B.orientalis-KF218819.1      -AAATAAGTTTTGCAAAACTTTAAGGTATGGTGAGACGACATGGAGGTGTCAATGAATTA
B.bovis-AB499088.1           AAAAAAAGTTTTGCAAAACTCTAAGGTATGGTGAGACGACATGGAGGTGTCAATGAATTA
                                * ***************  ******************************** *****

B.gibsoni-AB499087.1         ATTATATTAGAACTTTAATTAATTTTTAACCTGTTATCCCCGGCGTACCTTATTGCCAAT
B.bigemina-AB499085.1        ATTATATATGAACTACAATTAATTTTTAACCTGTTATCCCCGGCGTACCTTTTTGCCAAT
B.caballi-AB499086.1         ATTATATTAGAACTTTAATTAATTTTTAACCTGTTATCCCCGGCGTACCTTATTGCCAAT
B.orientalis-KF218819.1      ATTAAATAAGAACTTTAATTAATTTTTAACCTGTTATCCCCGGCGTACCTTATTGCCAAT
B.bovis-AB499088.1           ATTAAATTAGAGCTTTAATTAATTTTTAACCTGTTATCCCCGGCGTACCTTATTGCCAAT
                             **** **  ** **  *********************************** ********

B.gibsoni-AB499087.1         AATTGTAATTAGCTCTGATTAAATTTACGTAGAACTATTATATAACTTAATCTTCTTAAT
B.bigemina-AB499085.1        AATTGTAATTAGCTCTGATA-GATTTATGTAGAATCTTGATATTGCTTAATCTTCTCAAA
B.caballi-AB499086.1         AATGGTAATTAGCTCCGTAAAAATTTATGTAGAACTTTTATATTACCTAATCTTCTCAAA
B.orientalis-KF218819.1      AATTGTATTTAGCTCCATTACAATTTATGTAGAATTTTTATATAGCTTAATCTTCTTAAA
B.bovis-AB499088.1           AATAGTAATTAGCTCTGTTAAAATTTATGTAGAATCTCTATATAACTTAACCTTCTTAAT
                             *** *** *******       ***** ******     ****  * *** ***** **

B.gibsoni-AB499087.1         CATGAACTCATTCATTGCCAAACACGAATTTACTATACTTGAGTTATACAGTATTACCGC
B.bigemina-AB499085.1        CATGAACTCGTTCATTGCCAAACATGAATTTACTATACTTGGGTTATACAGTATTACCGC
B.caballi-AB499086.1         CATGAACTCGTTCATTGCCAAACACGAATTTACTATACTTGGGTTACACAGTATTACCGC
B.orientalis-KF218819.1      CATGAACTCGTTCAATGCCAAACATGAATTTATTATACTTGGGTTATACAGTATTACCGC
B.bovis-AB499088.1           CATGAACTCGTTCAATGCCAAACATGAATTTACTATACTTGGGTTATACAGTATTACCGC
                             ********* **** ********* ******* ******** **** *************

B.gibsoni-AB499087.1         GGCTGCTGGCACTGTATGATCTTACTATCTAATAACCAACTCTTATGTATGCTTGAACGC
B.bigemina-AB499085.1        GGCTGCTGGCACTGCATGATTTTACATATTAATATCCAACCCTTATGTATGCTTGAATGC
B.caballi-AB499086.1         GGCTGCTGGCACTGCATGATTTTACATATTGATATCCAACCCTTATGTATGCTTGAATGC
B.orientalis-KF218819.1      GGCTGCTGGCACTGTATGATTTTACATATCTATATCCAACCCTTATGTATGCTTGAATGC
B.bovis-AB499088.1           GGCTGCTGGCACTGTATGATTTTACATATTTATATCCAACCCTTATGTATGCTTGAATGC
                             ************** ***** ****      *** ***** **************** **

B.gibsoni-AB499087.1         TGTAATCAATAGTT--------TAAAGTTTTGAAATACTTTATGTAGTTATATATAATAA
B.bigemina-AB499085.1        TGTAACCAGAAATTTTTTAAAAAAAAGTTTTGAAATACTTTATGTAGATATATATAATAC
B.caballi-AB499086.1         TGTAATCAAAAATTGCTT----AAAAGTTTTGAAATACTTTATGTAGTTATATGTAATAC
B.orientalis-KF218819.1      TGTAATC---------------AAAAGTTTTGAAATACTTTATGTAGTTATATATAATAC
B.bovis-AB499088.1           TGTAATC---------------AAAAGTTATGAAATACTTTATGTAGTTATATATAATAC
                             ***** *                ****** ***************** ***** *****

B.gibsoni-AB499087.1         TGCTAGAATATTTAACGTCTAGTACTTAATAACGTTTAATATCAATTCCTACTTCCTTCA
B.bigemina-AB499085.1        TGCTAGAATATTTAACGTCTAGTACTTAATAACGTTTAATATCAATTCCTACTTCCTTCA
B.caballi-AB499086.1         TGCTAGAATATTTAACGTCTAGTACTTAATAACGTTTAATATCAATTCCTACTTCCTTCA
B.orientalis-KF218819.1      TGCTAGAATATTTAACGTCTAGTACTCATTAACGTTTAATATCAATTCCTACTTCCTTCA
B.bovis-AB499088.1           TGCTAGAATATTTAACGTCTAGTACTTAATAACGTTTAATATCAATTCCTACTTCCTTCA
                             ************************** * *******************************

B.gibsoni-AB499087.1         TCTTTACTCTTAAATATTTTAATAATATAATAACTAATAAATTCCTTCTAGAATTATGTT
B.bigemina-AB499085.1        TCTTTACTCTTAAATATTTTAATAATATAATAACCACGAAATTCCTTCTAGAATTATGTT
B.caballi-AB499086.1         TCTTTACTCTTAAATATTTTAATAATATAATAACCATTGAATTCCTTCTAGAATTATGTT
B.orientalis-KF218819.1      TCTTCACTCTTAAATATTTTAATAATATACTAACCAAGAAATTCCTTCTAGAATTATGTT
B.bovis-AB499088.1           TCTTCACTCTTAAATATTTTAATAATATATTAACCAATTAATTCCTTCTAGAATTATGTT
                             **** ************************ **** *   *********************

B.gibsoni-AB499087.1         AATTTGAGTAATGAAAATATCAGTATATTTAGTAACTTATTATAGGTCATTACTCTTCAT
B.bigemina-AB499085.1        AATTTGAGTAATGAAAAAATCAGTATATACAGTAACTTATTACAGGTCATTACTCTTCAT
B.caballi-AB499086.1         AATTTGAGTAATGAAAAAATCAGTATATACAGTAACTTATTACAGGTCATTACTCTTCAT
B.orientalis-KF218819.1      AATTTGAGTAATGAAAATATCAGTATATACAGTAACTTATTACAGGTCATTCCTCTTCAT
B.bovis-AB499088.1           AATTTGAGTAATGAAAATATCAGTATATGCAGTAACTTATTACAGGTCATTACTCTTCAT
                             ***************** **********  ************ ******** ********

B.gibsoni-AB499087.1         AAAAGATTTCACAGAGAACAATCTATAATTGTTCAAATTGGAATTTATCTGTTAACTTTA
B.bigemina-AB499085.1        AAAAGATTTCACAGAGAACAATCTATAATTGTTCAAATTGGAATTTATCTGTTAACTTTA
B.caballi-AB499086.1         AAAAGATTTCACAGAGAACAATCTATAATTGTTCAAATTGGAATTTATCTGTTAACTTTA
B.orientalis-KF218819.1      AAAAGATTTCACAGAGAACAATCTATAATTGTTCAAATTGGAATTTATCTATTAACTTTA
B.bovis-AB499088.1           AAAAGATTTCACAGAGAACAATCTATAATTGTTCAAATTGGAATTTATCTATTAACTTTA
                             ************************************************** *********

B.gibsoni-AB499087.1         TTTAATCCAACACCATTTTAAAGGTGTAAGGTTCATACCATAGTACTTAATAAAGTTAGA
B.bigemina-AB499085.1        TTTAATCCAACACCGTTTTAAGGGTGTGAGGTGCATACCAAAGTATTTAATAAAGTTAGA
B.caballi-AB499086.1         TTTAATCAAACACCGTTTTAAGGGTGGGAGGTTCATACCATTGTATTTAATAAAGTTAGA
B.orientalis-KF218819.1      TTTAATCAAACACCGTTTTAAGGGTGTGAGGTTCATACCAATATATTTAATAAAGTTAGA
B.bovis-AB499088.1           TTTAATCAAACACCGTTTTAAGGGTGGGAGGTTCATACCAGTGTATTTAATAAAGTTAGA
                             ******* ****** ****** ****  **** *******   ** **************

B.gibsoni-AB499087.1         TTACAATTTTTACTGAAATACTTAATTATATTAA-TTTATATCAGTGAGATATAGTGTGT
B.bigemina-AB499085.1        TTACAATTTTTACTGAAATACTTAATTATAGTAA-TTTATACCAGCAAGATATAGTGTGT
B.caballi-AB499086.1         TTACAATTTTTACTGAAATATTTAATTATATTAATTTTATATCAGTAAGATATAGTGTGT
B.orientalis-KF218819.1      TTACAATTTTTACTGAAATATTTAATTATATTAA-TTTATATCAGTAAGATATAGTGTGT
B.bovis-AB499088.1           TTACAATTCTTACTGAAATACTTAATTATATTAACTTTATATCAGTAAGATATAGTGTGT
                             ******** *********** ********* *** ****** ***  *************

B.gibsoni-AB499087.1         AGAATCAAATTAAACAACATGTTCCACTGACTTTACCACCTCTAACATTCCAATATGTAT
B.bigemina-AB499085.1        ATAATCAAATTAAACAACATGTTCCACTGATTTTACCACTTCTAACATTTCAATATGTAT
B.caballi-AB499086.1         ATATTCAAATTAAACAACATGTTCCACTGACTTTACCACTTCTAACATTTCAATATGTAT
B.orientalis-KF218819.1      ATAATCAAATTAAACAACATGTTCCACTGATTTTACCACTTCTAACATTTCAATATGTAT
B.bovis-AB499088.1           ATGATCAAATTAAACAACATGTTCCATTGACTCTACCACTTCTAACATTTCAATATGTAT
                             *   ********************** *** * ****** ********* **********

                                           mitoseqrev2
B.gibsoni-AB499087.1         GTTAAATATTAATTGACATCCAAGGCAAAGAATCGTAACACATTAATGTACTGATATTTA
B.bigemina-AB499085.1        GTTAAATATTTATTGACATCCAAGGCAAAGAATAGTAACACATTAATGTACTGGTATTTT
B.caballi-AB499086.1         GTTAAATATTTATTGACATCCAAGGCAAAGAATTGTAACACATTAATGTACTGATATTTT
B.orientalis-KF218819.1      GTAATATATATATTGACATCCAAGGCAAAGAATAGTAACACATTTATGTACTGATATTTT
B.bovis-AB499088.1           GTAAAATATATATTGACATCCAAGGCAAAGAATTGTAACACATTAGTGTACTGATATTTT
                             ** * ****  ********************** **********  ******* *****
```

```
B.gibsoni-AB499087.1        GGACAATTCTGACTTTGTGTAATTATATTATTTATACAAGTAAATGCATTTAAAATTAAT
B.bigemina-AB499085.1       GGATAATTCTGACTTTGTGTAATTATATAAAATATACAAGTTAATGTATTTAAAATTGAT
B.caballi-AB499086.1        GGATAATTCTGACTTTGTGTAATTATATAAAATATACAAGTTAATGTATTTAAAATTAAT
B.orientalis-KF218819.1     GGATAATTCTGACTTTGTGTAATTATATAAAATATACAAGTTAATGTATTTAAAATTTAT
B.bovis-AB499088.1          GGATAATTCTGACTTTGTGTAATTATAGTAAATATACAATTTAATGCATTTAAAATGAAT
                            *** ***********************  *  ******* * **** *********  **

B.gibsoni-AB499087.1        CTAACATCCAAATTTTAATCAATGATAGCGGTTAATCTTTCCTATTCCTTACGTACTCCG
B.bigemina-AB499085.1       CTAACATCCAAATTTTAAGCAAAGATAGCGGTTAATCTTTCCTATTCCTTACGTACTCCG
B.caballi-AB499086.1        CTAACATCCAAATTTTAAACAAAGATAGCGGTTAATCTTTCCTATTCCTTACGTACTCCG
B.orientalis-KF218819.1     CTAACATCCAAATTTTAATCAAAGATAGCGGTTAATCTTTCCTATTCCTTACGTACTCCG
B.bovis-AB499088.1          CTAACATCCAAATTTTAATCAATGATAGCGGTTAATCTTTCCTATTCCTTACGTACTCCG
                            ****************** *** *************************************

B.gibsoni-AB499087.1        GCTATGTTAATATTACTTTTGAAACAAGACTCTTAGATGCAAACTTCCCGGCTAAACTCC
B.bigemina-AB499085.1       GCTATATCAAATTTACTTTTGAAACAAGACTCTTAGATGCAAACTTCCCGGCTAAACTTC
B.caballi-AB499086.1        GCGATATCAAATTTACTTTTGAAACAAGACTCTTAGATGCAAACTTCCCGGCTAAACTTC
B.orientalis-KF218819.1     GCAATATCAAATTTACTTTTGAAACAAAACTCTTAGATGCAAACTTCCCGGCTAAACTCC
B.bovis-AB499088.1          GCTATATCAAATTTACTTTTGAAACAAAACTCTTAGACGCAAACTTCCCGGCTAAACTTC
                            ** ** * **  *************** ********* ******************** *

B.gibsoni-AB499087.1        CCTCTGTTTTATAAACGCATTCTAGCGCAATCCAATAGTTTAGTAACTGATACTAATATA
B.bigemina-AB499085.1       CCTCTTT-TTACAAGCGTATTCTTGAATAATCTAATAATTTGAATACAGCAATTAATATT
B.caballi-AB499086.1        CCTCTTTCTTACAAGCGTATTCTTGAACAATCCAATAATTTTACAACAGAAATAAACACT
B.orientalis-KF218819.1     CCTCTT--TTATATTCGAATTCTTGTACAATCTAATAGTTTTAATACACAGATTAAAACC
B.bovis-AB499088.1          CCTCTC--TTACATACGTATTCTGACACAATCTAATAACCTCATACAAGCATAAAGAACG
                            *****   *** *  ** *****     **** ****   *             *  *

B.gibsoni-AB499087.1        ATAAATATAGCACTTATACCTATAAACATAAGACCATCGTTTAAAGATAATCTTCCTAAA
B.bigemina-AB499085.1       AATATAATTGAGCATATTCCGATAAAAATTAAACCACTGTTTAATGATAATCTTCCTAGA
B.caballi-AB499086.1        AATATAATTGAACATACTCCAATGAACATAAGTCCACTGTTTAGCGATAATCTTCCTAAA
B.orientalis-KF218819.1     AGCGTTATTGAACAAATTCCTATGAATATTAATCCATTATTTAATGCCATCCTTCCTAAA
B.bovis-AB499088.1          ATTAAAACTGAACATATACCAATAAACATTAAACCATTGTTTAAAGCTAGTCTTCCAAGA
                            *     *  *  *  *  ** ** ** ** *  ***   ****  *  *  ***** * *

B.gibsoni-AB499087.1        CATCCTAGTATATATAATGCTGGAATCATAGGTATACTAATTATAGTCCATATTCTATGA
B.bigemina-AB499085.1       CTTCCCAATATAAATAATGCAGGTATCATTGGTATACTTATTAATGTCCATACTCTATGG
B.caballi-AB499086.1        CTTCCTAATACAAATAAAGCAGGAATCATAGGTATACTTATGATAGACCACATTCTGTGG
B.orientalis-KF218819.1     CTTCCAAGTATAATTAGAGCAGGAATCATAGGTATAGTCATAGAAACCCACATCCTGTGG
B.bovis-AB499088.1          CTTCCTAATATGAACAAAGCAGGTATCATAGGTAGAGTGATAGATGTCCACGATCTGTGA
                            * *** * **     *  ** ** ***** **** * * **      ***    ** **

B.gibsoni-AB499087.1        TAATGTACACATGATATTATAGGACTCATTGCTCTTGATTCAACAATTAAAATTAAAGAT
B.bigemina-AB499085.1       AAGTGTACACATGATATTACAGAGCTAGCAGCTCTTGATTCTACTATTAAAATCAAAGAT
B.caballi-AB499086.1        TAATGTACACAAGATATAACTGAGCTTGAAGCTCTTGATTCAACTATTAAAATCAAAGAT
B.orientalis-KF218819.1     TAGCTCGCACAGGATATGACTCTACTTATAGCCCTTGATTCCACTATTAGAATTATAGAT
B.bovis-AB499088.1          TACGTCACACAAGATACAACCGAACATATAGCTCTAGACTCTACTATTAAAATTATAGAT
                             *     **** ****  *     *     ** ** ** ** ** **** *** * ****

B.gibsoni-AB499087.1        TCTAATAATGCCGCCATAGCAATTAAACCTGCAAGTTTACTTGGGAATAATTTTAAAGTA
B.bigemina-AB499085.1       TCAAGTAGAAGACCCATAGCAATTAATCCAGCAATTTTACTTGGAAATAATTTTAGGGTT
B.caballi-AB499086.1        TCTAATAGAGCAGCCATAGCAATTAATCCTGCCATCTTACTTGGAAATAACTTCAAAGTT
B.orientalis-KF218819.1     TCTAATAGAATTCCCATAGCAATTAACCCTGCAATTTTACTTGGAAACAATTTCAGAGTA
B.bovis-AB499088.1          TCTAATAAAATTCCCATAGCAATTAATCCTGCCATCTTACTTGGGAACATCTTTAAAGTA
                            ** * **      ************* ** ** *  ******** ** *  ** *  **

B.gibsoni-AB499087.1        GCATAAAAAGTTAATAAATACCACTCTGGAACAATATGTAAAGGTGTTTGTAATGGGTTT
B.bigemina-AB499085.1       GCATAAAAAGTCAACAAATACCACTCTGGTACAATATGCATTGGTGTTTGCAAAGCGTTT
B.caballi-AB499086.1        GCATAGAAAGTTAATAAGTACCATTCAGGTACAATATGCATTGGTGTTTGAAGAGGATTT
B.orientalis-KF218819.1     GCGTAAAAAGTTAATAAATACCATTCTGGTACAATGTGCATAGGAGTTTGTAAAGGATTC
B.bovis-AB499088.1          GCATAAAAAGTTAATAGATACCATTCTGGTACTATATGCATTGGAGTTTGCAAAGGATTT
                            ** ** ***** ** *  ***** ** ** ** ** ** *  ** ***** *  *  **

B.gibsoni-AB499087.1        GATTCAATTGAATTATCAACATCTCCCTGAAACAATGGTATTATACCATAAGTTAATTGA
B.bigemina-AB499085.1       GCCATGATAGAATTATCACAGTCACCTTGGAACAATGGAATTACTCCATAAGTTAATTGA
B.caballi-AB499086.1        GACATGATAGAATTATCAACATCTCCTTGAAACAATGGAATAACTCCATATGTTAACTGA
B.orientalis-KF218819.1     GCCATGATTGAATTATCACAATCACCTTGAAACAAAGGTACTACTCCGTATGTCAATTGA
B.bovis-AB499088.1          GCCATAATTGAATTATCACAATCTCCTTGGAATAAAGGTATTACTCCATAAGTTAATTGA
                            *     ** *********   ** ** ** ** ** ** *  *  ** ** ** ** ***

B.gibsoni-AB499087.1        ACACCCAAAGCAGCAAATAGCATAGTCAACATCTTTAAATCACTGAATATTATAACAGGA
B.bigemina-AB499085.1       ACTCCAAGCAATATAAACAACATAGTCAACATTTTTATATCACTAAATATGATAACAGGA
B.caballi-AB499086.1        ACACCGAGTAAAATAAACAACATAGTTAACATTCTAATGTCACTAAATATAATAACAGGA
B.orientalis-KF218819.1     ATACCTAATATAATAAACAACATAGTTAACATCTTTATATCACTGAATATAATTACAGGA
B.bovis-AB499088.1          ACACCTAACAATATAAACAACATTGTTAGCATTCTAATATCACTGAATATAATTACTGGA
                            *  ** *       *** * *** ** * ***  * *  ***** ***** ** ** ***

B.gibsoni-AB499087.1        TAAAATCTTGATACATACCAAGAATCTACACCTGATAATGGATTTGTACTTGAAGATCTA
B.bigemina-AB499085.1       TAGAATCTAGAAACTAACCAAGAATCTATTCCAGATAATGGATTAGTACTTGAAGACCTG
B.caballi-AB499086.1        TAAAACCTAGAAACTAACCAAGAATCTATACCAGATAATGGATTAGTACTTGAGGACCTG
B.orientalis-KF218819.1     TAAAATCGTGAAACCAACCAAGAATCTATACCTGATAAAGGGTTGGTACTTGAAGATCTA
B.bovis-AB499088.1          TAAAATCTTGAAACTAACCAAGAATCTACTCCTGACAAAGGATTAGTACTTGAAGACCTG
                            ** ** *  ** **  ************  ** ** ** ** ** ******** ** **

B.gibsoni-AB499087.1        TGCAGATAATAAATATGAACTACTACTACACCCAATAATACAAAAGGTAAAATAAAGTGT
B.bigemina-AB499085.1       TGTAGATAATAAATGTGAATAATGACTATAAACAATAAAACGAAAGGCAATATAAAATGT
B.caballi-AB499086.1        TGCAAATAATAAATATGTATAATAACAACAAATAACAATACAAATGGTAAAATGAAATGT
B.orientalis-KF218819.1     TGCAAATAATAAATATGAATTAAAACAACGACCAACAAAACAAAAGGCAATATAAAATGA
B.bovis-AB499088.1          TGAAGGTAATAAATATGAATTAAAACAACAATTAACAATACAAAAGGTAATATGAAATGT
                            ** *  ******** ** *  *  ** *     ** ** ** ** ** ** ** ** **

B.gibsoni-AB499087.1        AATATATAAAATCTTTGTAAAGTTGGAACACTAACACTATAACCACCTAGTAGTACAATA
B.bigemina-AB499085.1       AGAATGTAGAACCTTTGTAATGTTGGGAATCCAACACCAAATCCTCCTAATACAAGAGAT
B.caballi-AB499086.1        AGTATATAAAACCTTTGCAAGGTTGGAAATCCTACACCATATCCTCCTAAAACAAGGACA
B.orientalis-KF218819.1     AGTATATAAAACCTTTGTAATGTAGGAAAGCCAACTCCGTAACCTCCAAGGACAAGAGGG
B.bovis-AB499088.1          AATATGTAAAATCTTTGTAGAGTTGGAAATGCAACTCCATAACCACCCAAAACAAGAGCG
                            *  ** ** ** ***** *  ** ** *     ** *   * ** ** *  *  *

                                                                             mitoR3
B.gibsoni-AB499087.1        ACAAAATCCGGAATCCAATAAAACAGGTTTGTTATAACTGTTGCTCCCCAATAACTCATT
B.bigemina-AB499085.1       ACCATATCAGGGAACCAGTAGAATAAATTAATTATAACTGTTGCTCCCCAGTAACTCATT
B.caballi-AB499086.1        ACCATATCTGGAAACCAATAAAACAAATTAATAATGACGGTAGCACCCCAGTAACTCATT
B.orientalis-KF218819.1     AGCATATCAGGAAACCAATAAAATAAATTAATTATAACTGTAGCTCCCCAATAACTCATT
B.bovis-AB499088.1          ACTAAATCTGGGAACCAGTAGAACAAGTTTATAATAACTGTTGCTCCCCAATAACTCATT
                            *  * *** ** * *** ** ** *  **  * ** ** ** ** ***** *********
```

```
B.gibsoni-AB499087.1         TGACCATTTGGTAAAACATAACCTAAGAATGCTATAGCCATACTTAAAACAAATATAGTC
B.bigemina-AB499085.1        TGTCCATTTGGAAGAACATAACCTAAGAAAGCAATTGCCATACTCAAAATAAATATAACC
B.caballi-AB499086.1         TGACCATTTGGAAGAACATATCCCAAAAAAGCTATAGCCATACTTAATATAAATATAACC
B.orientalis-KF218819.1      TGACCGTGTGGAAGAACATAACCTAAAAAGGCTATAGCCATACTCAATATAAATATAACC
B.bovis-AB499088.1           TGTCCGTAAGGAAGAACATAACCTAAGAAAGCAATAGCCATACTTAAAATAAATATAACC
                             ** ** *  ** * ****** ** ** ** ** ** ******** ** * *******  *

B.gibsoni-AB499087.1         ATTCCAGAATACCATGACCAAGGTAAATATTTACTTGAATACCATAATCCTTTTAACATA
B.bigemina-AB499085.1        ATTCCTGAATACCAAGACCAAGGTAAATGTTTACTTGAGTACCATAAACCTTTTAACATA
B.caballi-AB499086.1         ATTCCAGAATACCAAGACCAAGGTAAATACTTACTTGAATACCACAAACCCTTCAACATA
B.orientalis-KF218819.1      ATACCTGAATACCAGGACCAAGGTAAATACTTACTTGAATACCACAAACCTTTCAACATG
B.bovis-AB499088.1           ATTCCAGAATACCATGACCATGGTAGATATCTGCTTGAGTACCACAATCCCTTTAGCATA
                             ** ** ******** ***** **** **    * ***** ***** ** ** ** * ***

B.gibsoni-AB499087.1         TGTAAAAACATAAAGAAAAAACAGAAAGAAACACATTGAGCATGAAAGTAACGAACTGCC
B.bigemina-AB499085.1        TGTAACATCATAAAGAAAAAGCAAAAAGAAACACATTGAGCATGGAAATAACGAAGTGCC
B.caballi-AB499086.1         TGAAGCATCATAAAAAAGAAGCAAAAAGACACACACTGAGCATGAAAATAACGAAATGCC
B.orientalis-KF218819.1      TGTAACATCATAAAAAAGAAACAAAAAGAAACACATTGAGCATGAAAATAACGAAATGCC
B.bovis-AB499088.1           TGTAGCATCATGAAAAAGAAGCAAAAAGAAACACATTGAGCGTGAAAATAACGCATTGCC
                             ** *  * *** ** ** ** ** ***** ***** ***** ** ** ***** * ****

B.gibsoni-AB499087.1         CAACCAATATTAACTTCTGTTAATACTCTAATAACACTATTAAAAGATTCCATTCCTCCT
B.bigemina-AB499085.1        CAACCCATATTAACCTCAGTTAATACTCTGATAACACACTCGAAAGAATCAACATCTCCT
B.caballi-AB499086.1         CAACCCATATTAACCTCAGTTAATACTCTGATCACACATTCGAAAGATTCAACACCTCCA
B.orientalis-KF218819.1      CAACCCATATTAACTTCAGTTAAAACTCTAATAACACATTCAAAAGAATCAACATCTCCT
B.bovis-AB499088.1           CAACCCATATTGACTTCAGCAAGCACTCTAATTACACATTCAAAAGAATCAACGCTTCCA
                             ***** ***** ** ** *  *  ***** ** ****  *  ***** ** *    ***

B.gibsoni-AB499087.1         GGAATATAATAAAAAGTTAGCATCAGTCCAGACATTATCTGAAATACAAATACAAAACCC
B.bigemina-AB499085.1        GGAACATAATAAAAGGTCATAAGTAAACCAGAGAAAATCTGGAATACAAAAACGAAACCC
B.caballi-AB499086.1         GGTACATAATAGAAGGTTAAAAGCAAACCAGAAAATATCTGGAATACAAATACAAAACCT
B.orientalis-KF218819.1      GGAACATAATAAAAAGTCAAAAGAAGACCAGATAGTATTTGAAAAATAAATACAAAACCT
B.bovis-AB499088.1           GGAACATAATAAAATGTCAACAAAAGTCCTGATAGTATTTGAAATACAAATACAAAACCG
                             ** * ****** ** ** *  *  *  ** ** *  ** ** ** * *** ** *****

B.gibsoni-AB499087.1         ACTATAAATCCTAAATTCCAATTAGAATTTAAATTCTTTGGAACCAAATAGGACAACATA
B.bigemina-AB499085.1        ACGATAAAACCTAAGTTCCAATTAGAATTCAAATTCCTTGGAACTAAATAGGTTAGTAAA
B.caballi-AB499086.1         ACAATAAAACCTAAGTTCCAATTAGAATTCAAATTCCTTGGAACTAAATAGGATATCAAA
B.orientalis-KF218819.1      ACTATAAATCCCAAATTCCAATTAGAATTCAAATTCCTTGGAACAAGATAGGAAAGCAAA
B.bovis-AB499088.1           ACTATAAACCCTAAGTTCCAATTAGAATTCAAATTCCTTGGAACAAGATAGGAAAGTAGA
                             ** ***** ** ** ************** ****** ******* * *****  *  * *

B.gibsoni-AB499087.1         TGAGAATTAAATAAATTTAATGCAAATGTTAACTTGTTGGTTAAAGCCCTGTTTCCTAAA
B.bigemina-AB499085.1        TGAGAATTAATCAAATTCATTGCAAATGTTAACTTGTTGGTTAAAGCCCTGTATCCTAAA
B.caballi-AB499086.1         TGAGAGTTTACTAAATTCATTGCAAATGTTAACTTGTTGGTTAAAGCCCTGTTTCCTAAA
B.orientalis-KF218819.1      TGAGAATTTACTAAATTCATTGCAAATGTTAATTTGTTGGTTAAAGCCCTATATCCTAAA
B.bovis-AB499088.1           TGAGAGTTTAACAAATTCATTGCAAATGTTAACTTGTTGGTTAAAGCCCTGTATCCTAAA
                             ***** ** *  ***** * ************ **************** * *******

B.gibsoni-AB499087.1         CATTTTTCAATATAACATATATATATAATATTTATACCTGTCAAGTTCCTTCACTAAATT
B.bigemina-AB499085.1        ATCGATCTTATATAACAATACTATAAAAATTTTATACCTGTCAAGTTCCTTCACTAAATT
B.caballi-AB499086.1         TTT--ATCTTTATAACAAACATTAATAAATTTTATACCTGTCAAGTTCCTTCACTAAATT
B.orientalis-KF218819.1      CTT--ATATATATAACA-ATGTAAATAAATTTTATACCTGTCAAGTTCCTTCACTAAATT
B.bovis-AB499088.1           CTT--TTTTATATAACATAATCAAATAAATTTTATACCTGTCAAGTTCCTTCACTAAATT
                                       *******       * **  ******************************

B.gibsoni-AB499087.1         TTACTCACAGTATAGTAATACATTATTAACGTGATTTCAACAATGGTGATAAAAATTTAA
B.bigemina-AB499085.1        TCTCTCACAGTATAGAAATATATTTTTAACGTGATTTCGACAATGGTGTTAAATATTTAA
B.caballi-AB499086.1         TCTCTCACAGTATAGAAATATATTTTTAACGTGATTTCAACAATGGTGTTAAACATTTAA
B.orientalis-KF218819.1      TCTCTCACAGTATAGAAATATATT-TTAACGTGATTTCAACAATGGTGTTAAACAATTAA
B.bovis-AB499088.1           TCTCTCACAGTATAGAAATATATT-TTAACGTGATTTCAACAATGGTGTTAAACAATTAA
                             *  ************ **** *** ************* ********* **** * ****

B.gibsoni-AB499087.1         CGCTTGAATAAGGTTTAGTAGGTATGAGAGTTCATCATATTAATATGGTACGTGAGTTGG
B.bigemina-AB499085.1        TGCTTGAATAAGGTTTAGTAGGTATGAGAGTTCATCATATTAATATGGTACGTGAGTTGG
B.caballi-AB499086.1         TGCTTGAATAAGGTTTAGTAGGTATGAGAGTTCATCATATTAATATGGTACGTGAGTTGG
B.orientalis-KF218819.1      TGCTTGAATAAGGTTTAATAGGTATGAGAGTTCATCATATTAATATGGTACGTGAGTTGG
B.bovis-AB499088.1           TGCTTGAATAAGGTTTAATAGGTATGAGAGTTCATCATATTAATATGGTACGTGAGTTGG
                              **************** ******************************************

B.gibsoni-AB499087.1         GTTAAGAACGCTGAGAAGCAGTTCGTTCCCTATCTAGTATATTTAAAATTAATATAACAA
B.bigemina-AB499085.1        GTTAAGAACGCTGAGAAGCAGTTCGTTCCCTATCTAGTATATTT-AACTTAACATAATAT
B.caballi-AB499086.1         GTTAAGAACGCTGAGAAGCAGTTCGTTCCCTATCTAGTATATTT-AATTCAATATAAT--
B.orientalis-KF218819.1      GTTAAGAACGCTGAGAAGCAGTTCGTTCCCTATCTAGTATATTT-AAATTTAAATAATAA
B.bovis-AB499088.1           GTTAAGAACGCTGAGAAGCAGTTCGTTCCCTATCTAGTATATTT-AAATCTATATAAT--
                             ******************************************** ** *  * ****

B.gibsoni-AB499087.1         TAAACAACTTAAAACTTTTTTTAAAAAAATACAA----------CTTAATAAAACTAATA
B.bigemina-AB499085.1        CTTTTCTTAAAAAACTTTATTAAAAAACACTAACAA--ACAACTCAAAGCAAAGCTGACA
B.caballi-AB499086.1         --TTTCAAAAAAAACTTTTTTTACAAAAAAATAAAGAAATACTTTCAAA-----------
B.orientalis-KF218819.1      TACTT---AAAAACTTTTAACAATAAACAAAAAAAACAGAAAATTTCAACAAATTTGAAA
B.bovis-AB499088.1           --TTTGAAAAAAAACTTTATAACACAACACAAAAAA---CATATTTCAAAAATTTTGAAA
                                       ***  ***       ** *   *              *

B.gibsoni-AB499087.1         AAAAATTCTCTTTTATTAA-----------------------------------------
B.bigemina-AB499085.1        GTTATTCGTTCAGTGTTAATTGTAT-GTAACAAGTGTCCTTTATACATGATCACTTGTC-
B.caballi-AB499086.1         -------------TGGTGG-----------------------------------------
B.orientalis-KF218819.1      TTCATCAGTTTT-TGTTATTTAACT-GTAACAAGTGTACTTTATACATGATCACTTGTTT
B.bovis-AB499088.1           TTAAATTGTTTT-TGATATTTACACAGTAACAAGTGTACTTTATACATGATCACTTG---
                                          *  *

B.gibsoni-AB499087.1         ------------------------------
B.bigemina-AB499085.1        ------------------------------
B.caballi-AB499086.1         ------------------------------
B.orientalis-KF218819.1      AAATTTCAACAAATATAAAGTTTTTTAACA
B.bovis-AB499088.1           ------------------------------
                                            mitoR5
```

Figure S1: Alignment of mitochondrial genomes from various *Babesia* species, with primer binding sites highlighted in red. Conserved regions are marked with asterisks (*), indicating high sequence similarity across species.
